# Suitability of Different Diagnostic Platforms for Virological Testing of Blood Samples from Cornea Donors

**DOI:** 10.1159/000524250

**Published:** 2022-06-14

**Authors:** Niko Kohmer, Marhild Kortenbusch, Annemarie Berger, Cornelia Rühl, Sandra Ciesek, Sabine Salla, Holger F. Rabenau

**Affiliations:** ^1^Institute for Medical Virology, University Hospital, Goethe University Frankfurt am Main, Frankfurt, Germany; ^2^German Centre for Infection Research, External Partner Site Frankfurt, Frankfurt, Germany; ^3^Fraunhofer Institute for Molecular Biology and Applied Ecology (IME), Branch Translational Medicine and Pharmacology, Frankfurt, Germany; ^4^Department of Ophthalmology, RTWH Aachen University, Aachen, Germany

**Keywords:** Donor screening, Virological testing, Serology, PCR

## Abstract

**Background:**

To minimize the risk of disease transmission in cornea transplantation, donor screening for blood-derived viral infections is mandatory. Ideally, pre-mortem blood samples are used, but based on availability, cadaveric blood samples of cornea donors may also be used. However, serological and nucleic acid amplification tests (NATs) need to be validated for the use of cadaveric specimens.

**Methods:**

Hepatitis B virus (HBV), hepatitis C virus (HCV), human immunodeficiency virus (HIV), human T-lymphotropic virus (HTLV) 1/2, and *Treponema pallidum* (syphilis)-specific serological and/or NAT assays were validated on different platforms (Abbott Alinity i, Alinity m, Roche Cobas 6800, and Roche Cobas AmpliPrep/Cobas TaqMan (CAP/CTM)) using (un)spiked paired pre- and post-mortem cornea donor blood samples from the same individual (up to 23.83 h after death) of 28 individuals in accordance with the specifications of the German Federal Institute for Vaccines and Biomedicines (Paul-Ehrlich-Institut [PEI]). In addition, routinely HBV-, HCV- and HIV-PCR-negative tested post-mortem blood samples of 24 individuals were used to assess NAT specificity.

**Results:**

For the majority of serological parameters on the Abbott Alinity i (HBsAg, anti-HBc, anti-HBs, anti-HCV, anti-HIV, anti-HTLV 1/2, and anti-*Treponema pallidum*), ratios of generated test results of (un)spiked paired pre- and post-mortem blood samples differed ≤25%, with an agreement of qualitative pre- and post-mortem test results ranging from 91.2 to 100%. For NAT parameters (HBV, HCV, and HIV) on the Cobas 6800, Alinity m, and CAP/CTM, no significant deviation in virus concentrations (factor >5) of spiked pre- and post-mortem blood samples could be observed. Ct-values of corresponding internal controls did also not differ significantly (>1.5 Ct-values). In addition, no false-positive test results were generated when specificity was assessed.

**Conclusion:**

Overall, fluctuations of test results for serological and NAT parameters in pre- and post-mortem blood samples examined in this study, were only limited and within the range of what is also observed when routinely testing fresh patient specimens. We conclude that all examined assays are eligible for the screening of blood samples taken up to about 24 h after the occurrence of death.

## Introduction

Among allogenic human tissue transplantations, corneal transplantation is the most frequently performed type of transplant across the world [1]. To minimize the risk of potential disease transmission, as regulated by EU and accompanying national directives, tissue donor screening for blood-derived viral infections is mandatory [2, 3]. Ideally, pre-mortem blood samples are used for screening, but also cadaveric blood samples of cornea donors may be obtained until maximum 24 h post-mortem. However, haemolysis, autolysis, and other factors may interfere with assay sensitivity and specificity with the risk of generating false-negative or false-positive test results [4]. To ensure routinely used tests meet the specified quality requirements when testing post-mortem blood samples, they need to be validated [5, 6, 7]. But the majority of commercially available screening assays are not or only validated for the use of cadaveric blood samples in a limited manner [8]. With the introduction of newly available assays and testing platforms, this list continues to grow. Therefore, focusing on paired pre- and post-mortem cornea donor blood samples from the same individual, the latter taken within the full legal timeframe of up to about 24 h post-mortem, we validated a variety of routinely used hepatitis B virus (HBV), hepatitis C virus (HCV), human immunodeficiency virus (HIV), human T-lymphotropic virus (HTLV) 1/2, and *Treponema pallidum* (*T. pallidum*, syphilis)-specific serological and/or nucleic acid amplification test (NAT) assays on different platforms: Abbott Alinity i, Alinity m, Roche Cobas 6800, and Cobas AmpliPrep/Cobas TaqMan (CAP/CTM). Validation was carried out in compliance with the specifications of the German Federal Institute for Vaccines and Biomedicines (Paul-Ehrlich-Institut [PEI]) [9, 10]. Selected serological and NAT assays examined in our study have already been validated and approved by the test manufacturer (Abbott) for the use of post-mortem blood samples, however only for a maximum timeframe between occurrence of death and blood collection between 15 and 21.5 h. This results in a time delta (ΔT) to the permitted time limit of 24 h and therefore a loss of acceptable cornea donations in cases where the upper timeframe not validated by the manufacturer is exceeded. In addition, some assays have not been validated for the use of cadaveric specimens at all (e.g., anti-HBs). Our study results complete the assay portfolio by also extending the timeframe between occurrence of death and blood collection up to 23.83 and 23.33 h for serological and NAT parameters, respectively.

## Materials and Methods

### Blood Sampling

Pre- and post-mortal blood samples of 28 cornea donors were prospectively collected under standardized conditions, fulfilling all criteria of the PEI specifications. According to legal requirements, a haemodilution of more than 50% before blood sampling was ruled out. Pre-mortem samples were collected at a maximum of 7 days before death. Cadaveric blood samples of 19 individuals (serum and/or EDTA plasma) were collected between 17.58 and 23.83 h for the validation of serological parameters and of 9 individuals for NAT validation between 16.18 and 23.33 h after death (online suppl. Table S1, S2; for all online suppl. material, see www.karger.com/doi/10.1159/000524250). In addition, 24 on the already validated Roche CAP/CTM system routinely HBV, HCV and HIV PCR negative tested post-mortem samples (Hornhautbank Aachen, University Hospital RWTH Aachen; Deutsche Gesellschaft für Gewebetransplantation) taken up to 24 h after the occurrence of death were used to assess NAT specificity. Specimens were centrifuged within 1 h after blood withdrawal and stored at −30°C until further analysis was performed. During the test phase, samples were stored at 4–8°C, which corresponds to the storage conditions in the routine laboratory procedure as well as to the test manufacturer's specifications.

### Sample Preparation

For the validation of serological parameters, samples were spiked using the PEI standards “HIV-Referenz IV” with a dilution of 1:100,000 (low level) and 1:200 (high level), “HCV-IgG Reference Serum” with a dilution of 1:200 (low level) and 1:10 (high level), “HBsAg subtype Ad, 100 U/mL” with a dilution of 1:2,000 (low level) and 1:200 (high level) as spiking reference samples. For the parameters anti-HBc, anti-HBs, anti-*T. pallidum*, and anti-HTLV 1/2, routinely positive tested samples were diluted and used for spiking. For anti-HBc, a patient sample (S/CO 10.67) was diluted 1:26,500 and 1:50, resulting in a low- and high-level spiking reference sample. For anti-HBs, a sample containing 290 U/mL was diluted 1:20 or 1:5; for anti-*T. pallidum*, a sample (S/CO 19.06) was diluted 1:328 or 1:50; and for anti-HTLV 1/2, a sample (S/CO 162) was diluted 1:8,000 or 1:50 in order to generate low- or high-level spiking reference samples.

For the validation of the NATs, samples were spiked with routine patient samples measured by the already validated CAP/CTM containing either HBV (initial concentration of 152,690 IU/mL), HCV (initial concentration of 2,480.000 IU/mL), or HIV (initial concentration of 1,546.195 HIV-RNA copies/mL) targeting the 3-fold (or 15-fold, when also considering the routinely used 1:5 predilution) limit of detection (LOD) of the NAT validated for the normal serum/plasma samples according to the PEI specifications. However, the theoretical concentration was marginally exceeded in most of the cases, for example, for plasma on the Cobas HBV assay and according to manufacturer specifications: theoretical 40.5 IU/mL, in fact 51 IU/mL, for the Alinity m theoretical 105 IU/mL, in fact 208 IU/mL, for the CAP/CTM theoretical 300 IU/mL, in fact 57 IU/mL. The spiking experiments resulted in mean HCV-RNA concentrations of 289, 189, and 338 IU/mL, HBV-DNA concentrations of 51, 208, and 57 IU/mL, and HIV-RNA concentrations of 145, 143, and 165 copies/mL for the Cobas 6800, Alinity m, and CAP/CTM, respectively. To prevent potential inhibition, all samples were prediluted 1:5 with human blood plasma, which was negative for all three parameters, prior to further testing.

### Serological Tests

The Abbott anti-HBc II, anti-HBs, HBsAg qualitative II, HBsAg qualitative II confirmatory, anti-HCV, HIV Ag/Ab Combo, rHTLV-I/II, syphilis TP reagent kits, and corresponding calibrators and controls were used on the Abbott Alinity i platform according to the manufacturer's recommendation. A sample volume of 200 μL and depending on the dilution 1.5–20 μL (only for anti-HBs a maximum of 40 μL) of spiking material were used. Serological parameters were validated and approved by Abbott for analysis of post-mortem blood samples and maximum timeframe (in hours) between occurrence of death and blood collection: anti-HBc II (17.5 h), anti-HBs (not validated), HBsAg qualitative II, (18.5 h), HBsAg qualitative II confirmatory (not validated), anti-HCV (15 h), HIV Ag/Ab (17.5 h), rHTLV-I/II (18.5 h), and syphilis TP (21.5 h).

### Nucleic Acid Amplification Tests

The Abbott HIV-1 AMP Kit, HBV AMP Kit, and HCV AMP Kit were used on the Abbott Alinity m platform according to the manufacturer's recommendation. For the Roche Cobas® 6800, the HIV-1, HBV, and HCV assays and for the Roche CAP/CTM the HIV Quantitative Test Version 2.0 and HBV/HCV Test Version 2.0 were used. All tests were carried out according to the manufacturer's recommendation. The used sample volumes for each analysis were as follows: 2.5 mL for the Abbott Alinity m and Cobas® 6800 platforms and 1 mL (HIV Quantitative Test Version 2.0) or 750 μL (HBV and HCV Quantitative Test Version 2.0, each) for the Roche CAP/CTM.

## Results

### Serological Parameters

For the validation of serological parameters on the Alinity i, paired pre- and post-mortem blood samples (serum and plasma) of 19 individuals were analysed (online suppl. Table S3–S5). To check reproducibility in the lower and upper test range, samples were spiked according to the PEI specifications resulting in low- and high-level positive samples [9]. Because sample volumes were limited, varying numbers of analyses were carried out (Table 1). Qualitative agreement of pre- and post-mortem test results is shown in Table 2.

The highest agreement of 100% between pre- and post-mortem test results was achieved for the anti-HCV, anti-HIV, anti-HTLV 1/2, and anti-*T. pallidum* assays, followed by the anti-HBc, anti-HBs, and HBsAg assays with an agreement of 98.4%, 96.2%, and 91.2%, respectively. Regarding the discrepancy of qualitative HBsAg test results, in 6 out of 68 analyses (8.8%), a negative result was detected in the pre-mortem serum sample, while the post-mortem sample was found to be weakly reactive (S/CO between 1.45 and 4.59), which could not be verified in the confirmation test (neutralization test). For anti-HBc, in 1 of 64 analyses (1.6%) a weakly reactive result (S/CO = 1.78) was detected in the pre-mortem serum sample, while the post-mortem sample proved to be negative (S/CO = 0.74), and for anti-HBs in 2 of 53 analyses (3.8%), a weakly reactive result (mIU/mL = 12.58 and 11.86, respectively) was detected in the pre-mortem taken serum and EDTA plasma samples, while the samples taken post-mortem were tested negative.

**Fig. 1:**
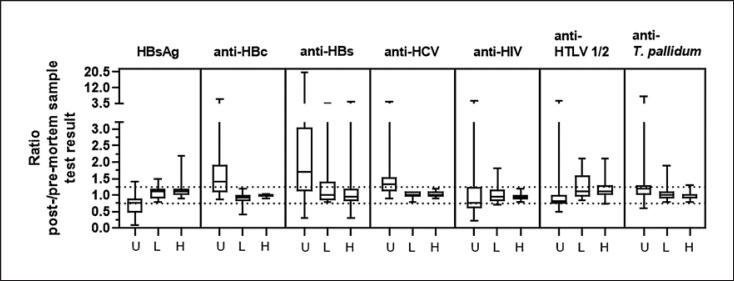
Range of post-/pre-mortem sample test result ratios. Boxes represent the interquartile range including median, upper, and lower whiskers showing minimum and maximum values. U, unspiked; L, low spike level; H, high spike level; dotted horizontal lines indicate the upper +25% and lower −25% tolerance limit.

Ratios of generated test values between cadaveric and normal samples, irrespective of the matrix used (serum or plasma), are expressed in Figure 1. According to the specifications of the PEI, test results (either S/CO value or mIU/mL) of the paired samples should not differ more than 25% for positive-spiked samples.

The distribution of post- and pre-mortem test result ratios, irrespective of used matrix (serum or plasma), for unspiked (U), and low (L)- and high (H)-level spiked samples for each examined assay, is shown in Table 3. For the majority of the sample pairs analysed, ratios of test values of pre- and post-mortem blood samples differed ≤25%. The majority of deviations were observed in the result ratios of negative (unspiked) samples, which are not part of the PEI requirements.

Overall, fluctuations of test values in different pre-mortem samples from the same donor (serum or EDTA plasma), but also between the pre- and post-mortem blood samples, were only limited and within the range of what is also observed when routinely testing fresh patient specimens and evaluating the intra- and interassay reproducibility. A more detailed analysis of the serological parameter test results can be found in the online supplementary material.

### Nucleic Acid Amplification Tests

**Fig. 2:**
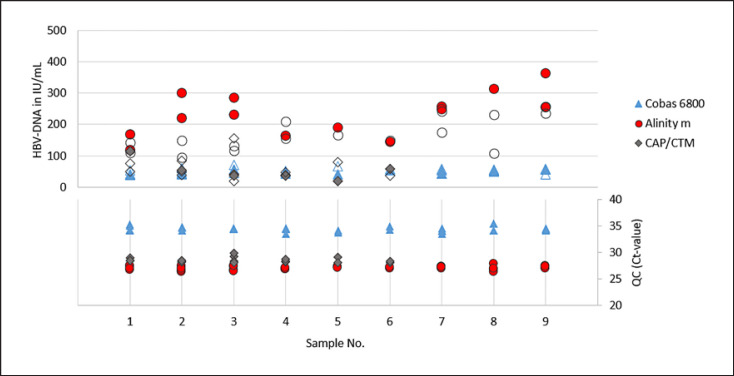
Quantitative HBV-DNA concentrations (in IU/mL) observed in spiked and 1:5 prior testing diluted pre- and post-mortem blood samples (irrespective of used matrix, serum, and plasma) of 9 cornea donors on each test system and the corresponding Ct-values of the internal QCs. Filled symbols (quantitative depiction) = samples taken post-mortem, empty symbols = samples taken pre-mortem (in each case not differentiated between serum and EDTA plasma).

**Fig. 3:**
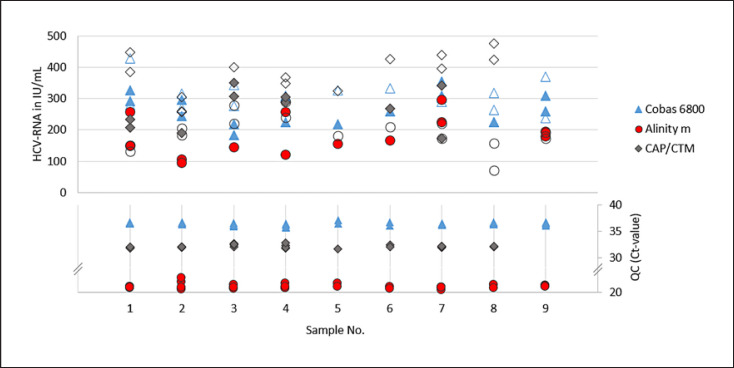
Quantitative HCV-RNA concentrations (in IU/mL) observed in spiked and prior testing 1:5 diluted pre- and post-mortem blood samples (irrespective of used matrix, serum, and plasma) of 9 cornea donors on each test system and the corresponding Ct-values of the internal QCs. Filled symbols (quantitative depiction): samples taken post-mortem, empty symbols: samples taken pre-mortem (in each case not differentiated between serum and EDTA plasma).

**Fig. 4:**
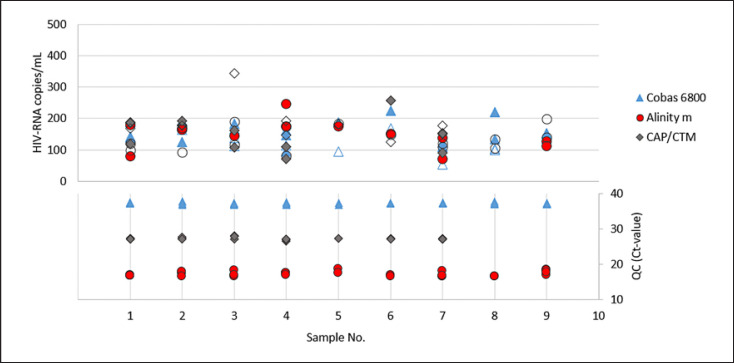
Quantitative HIV-RNA concentrations (in copies/mL) observed in spiked and prior testing 1:5 diluted pre- and post-mortem blood samples (irrespective of used matrix, serum, and plasma) of 9 cornea donors on each test system and the corresponding Ct-values of the internal QCs. Filled symbols (quantitative depiction) = samples taken post-mortem, empty symbols = samples taken pre-mortem (in each case not differentiated between serum and EDTA plasma).

For the validation of the NATs in the detection of positive specimens, paired pre- and post-mortem blood samples (serum and plasma) of 9 cornea donors were used. To check assay reproducibility in the lower LOD range, samples were spiked with the respective virus (HBV, HCV, and HIV) at 3-fold LOD of the NAT validated for the normal serum/plasma samples according to the PEI specifications. For HCV, spiked pre- and post-mortem serum (*n* = 9) and plasma (*n* = 7) pairs were analysed on all three test systems. Same sample pairs were used for HBV and HIV on the Cobas 6800 and Alinity m. Because of limited sample volume, only 7 spiked pre- and post-mortem serum, and 4 to 5 plasma pairs were used on the CAP/CTM for HBV and HIV, respectively. In one case only pre-mortem serum (HBV and HIV) and in two cases (HIV) only post-mortem sera were tested. To prevent potential inhibition, samples were diluted 1:5 prior testing (online suppl. Table S6–S8). This was taken into consideration when determining the spike amount. Quantitative HBV-, HCV-, and HIV-PCR results and corresponding internal controls for each test system are shown in Figures 2, 3, 4 and summarized characteristics in Table 4.

In the quantitative determination of HBV-, HCV-, and HIV-PCR virus concentrations of spiked, pre- and post-mortem serum, and plasma samples from cornea donors, no significant deviation (factor >5) was observed on any of the three test systems examined (Cobas 6800, Alinity m, and CAP/CTM). Deviations in virus concentrations ranged from a factor of 1.5 to 2.9 for HBV-DNA, from 1.6 to 2.7 for HCV-RNA, and from 2 to 2.6 for HIV-RNA. In addition, the Ct-values of the corresponding internal controls did not differ significantly (deviations >1.5 Ct-values from the mean were defined as significant).

To check assay specificity and potential negative impact of cadaveric specimens on the internal quality controls (QCs), 24 HBV-, HCV-, and HIV-PCR-negative tested post-mortem blood samples were analysed (online suppl. Table S9–S11). No false-positive test results were generated. In addition, no negative influence on the HCV-, HBV-, or HIV-PCR results of the (1:5 diluted) post-mortem blood samples could be observed. Ct-values of the internal QC of the analysed specimens did not differ significantly from those of the negative control included in the test kit. There were also no significant deviations of individual Ct-values from the mean value of the 24 samples.

## Discussion

Ideally, cornea donor blood samples should be taken just before or within 7 days before death, as post-mortem blood samples harbour the potential risk of generating false test results when screening for blood-derived viral infections. Among other factors, this might be due to haemolysis, autolysis, bacterial contamination, or too late centrifugation [2, 11]. However, in some cases there are only post-mortem taken blood samples available and thus virological screening assays need to be validated for this sample type. Therefore, in our study, we validated a variety of routinely used serological and NAT parameters according to internal and PEI specifications using paired (un)spiked pre- and post-mortem blood samples from cornea donors, and the latter were taken up to about 24 h after death. All examined serological parameters on the Abbott Alinity i met the required specifications of the PEI. This is an interesting finding, as it has to be pointed out that the anti-HBs and HBsAg qualitative II confirmatory assays have not been validated for the use of cadaveric blood samples at all. We perceived the validation of anti-HBs as valuable in order to detect donors with an isolated anti-HBc, harbouring a potential risk for HBV transmission due to occult HBV infection. The anti-HTLV 1/2 assay was validated for donors with a corresponding risk profile. Also, for this and the other parameters, there are no data for specimens exceeding the post-mortem timeframe already validated by the manufacturer. In our study and based on the observed assay, the manufacturer-validated timepoints could be extended by additional 2.33–8.83 h post-mortem. If we could not use the full timeframe of about 24 h post-mortem, approximately 30% of cornea donations would have been needed to be discarded (consultation with the Department of Ophthalmology, RTWH Aachen University, Aachen, Germany).

Six HBsAg analyses showed positive test results for unspiked post-mortem samples, whereas the subsequently performed confirmatory assay was negative. In this case, false positivity was most likely caused by a post-mortem process such as autolysis, which resulted in a discrepancy of the qualitative serological agreement for 8.8% of the analyses. In one case, a weakly positive anti-HBc result was generated in a pre-mortem sample, whereas the post-mortem sample was tested negative. It is unknown whether this is a problem with assay specificity in the pre-mortem sample or a sensitivity problem in the post-mortem sample. For anti-HBs, two samples showed significantly higher test result ratios between pre- and post-mortem samples. Since this was observed in parallel in both the serum and the EDTA plasma sample, it can be assumed that this is not test-related, but probably caused by an intermediate medical intervention.

Comparable serological results were reported in different studies where post-mortem cornea donor blood samples taken up to about 24 h after the occurrence of death were validated. In one study by Baleriola et al. [12] and another from Kok et al. [13], non-related spiked and unspiked samples in variable numbers were validated using the HIV Ag/Ab, HBcAb, HBsAg, HTLV 1/2, anti-HCV, and *T. pallidum* assays on the Abbott Architect, the predecessor platform of the Alinity i. No significant differences between test results could be detected [12, 13].

One strength of our study is that paired spiked and unspiked pre- and post-mortem blood specimens were tested. This also applies to a study by Schmack et al. [7] where similar results were generated when this platform was used and screened for HBsAg, anti-HBc, anti-HCV, anti-HIV-1/2, and HIV p24 Ag. They tested 20 related spiked and unspiked pre- and post-mortem blood on the Architect, and no false-positive or false-negative results were generated. However, 1 weak signal in a post-mortem sample for anti-HIV-1 and HIV-1 p24 Ag was detected [7]. False positivity was observed for anti-*T. pallidum* in a study by Kalus et al. [14] on a Siemens BEP-III, where 2 out of 10 related and unspiked post-mortem sera taken up to 58 h post-mortem were false-positive, most likely due to intense haemolysis. In the validation of 20 spiked post-mortem blood samples taken up to 52 h post-mortem on the Siemens BEP-III, anti-HIV 1/2, anti-HCV, HBsAg, and anti-HBc were correctly detected [15]. Despite the suitability of validated assays for the serological screening of cadaveric samples, in a study by Larscheid et al. [16] the total IgG in post-mortem blood samples of tissue donors was significantly lower when compared to pre-mortem blood samples. This highlights the utility of additional NAT-based screening for the confirmation of negative, equivocal, or weakly positive test results, thus increasing the safety of tissue preparations [17, 18]. Regarding the NAT-based assays validated in our study, all examined assays were able to detect all HBV, HCV, and HIV spiked and unspiked pre- and post-mortem samples with no significant deviations of virus concentrations and internal QCs. In addition, all negative samples were identified correctly with valid QC, indicating no inhibition occurred due to the use of cadaveric samples. This might be due to preventive 1:5 predilution of each sample to avoid inhibitory interference. This is in accordance with a validation study from Gubbe et al. [5] where the DRK Baden-Württemberg-Hesse CE PCR kits for HBV, HCV, and HIV were validated for the use of cadaveric blood samples on their own NAT system. The assays demonstrated a sensitivity and specificity of 100% when unrelated spiked and unspiked pre- and post-mortem blood samples (*n* = 72) taken up to 24 h after the occurrence of death were tested [5]. In the study by Schmack et al. [7], also no significant deviations could be observed when performing HCV-PCR on an Abbott m2000rt cycler using 20 related spiked and unspiked pre- and post-mortem cornea donor blood samples, the latter taken up to 24 h after death. In a study by Ribeiro et al. [6], the Roche CAP/CTM HIV-1 Test, v2.0, demonstrated a sensitivity and specificity of 100%, whereas in contrast to our study, the CAP/CTM HCV Test, v2.0, only demonstrated a sensitivity of 80% and specificity of 60% when testing 20 spiked and 5 unspiked post-mortem blood samples with a timeframe between death and blood sampling <24 h.

Post-mortem samples may be used up to a maximum of 24 h after the occurrence of death, because they are often of inferior quality. Macroscopically abnormal cadaveric serological samples should be interpreted with care [19]. In addition, haemodilution and other factors also need to be assessed when screening is performed [17]. Studies showed that the timeframe of post-mortem taken blood samples may be further extended >24 h with serological and NAT assays still generating valid test results [5, 15, 20, 21]. Regarding sample storage, continuous stability of HCV-RNA in plasma samples from cornea donors stored at 4°C for up to 8 days and of HBsAg, anti-HBc, and anti-HCV stored in frozen (−20°C/−70°C) dried blood spots and eluates for up to 200 days has been reported [22, 23]. However, according to European (EDQM) and German guidelines, corneas must be explanted within 72 h after death, but donor blood has to be taken within a maximum of 24 h post-mortem.

One advantage of our sample constellation is that not only unspiked and spiked post-mortem blood samples, but also pre- and post-mortem samples from the same donor were used. Thus, individual donor-specific factors as well as matrix-dependent factors were taken into account.

With regard to our study, we can summarize that fluctuations of test results for serological and NAT parameters in pre- and post-mortem blood samples examined in this study were only very limited and within the range of what is also observed when routinely testing fresh patient specimens. No significant deviations according to internal and PEI specifications could be observed. Validation data of our study suggest that all examined assays are eligible for the screening of post-mortem blood samples taken up to about 24 h after death.

## Statement of Ethics

Ethical review and approval were waived for this study as testing of already existing specimens is already covered by an ethics vote.

## Conflict of Interest Statement

Sandra Ciesek received research support and speaker's fee from Roche diagnostics. All other authors have no conflict of interest to declare.

## Funding Sources

No specific funding for this study was received.

## Author Contributions

Conceptualization, methodology, and formal analysis: Holger F. Rabenau, Niko Kohmer, and Annemarie Berger; resources: Holger F. Rabenau and Sabine Salla; experiments: Marhild Kortenbusch and Cornelia Rühl; supervision: Holger F. Rabenau and Sandra Ciesek; original draft preparation: Niko Kohmer and Holger F. Rabenau; all authors reviewed and edited the manuscript.

## Data Availability Statement

Data supporting the findings of this study are available within the article and its online supplementary material. For further information, please contact the corresponding author.

## Supplementary Material

Supplementary data

## Figures and Tables

**Table 1:** Number of tested pre/-post-mortem sample pairs* of either serum or plasma in each examined serological assay

Material	Tested pre-/post-mortem sample pairs in each assay, *n*							
	HBsAg	anti-HBc	anti-HCV	anti-HIV	anti-*T. pallidum*	anti-HTLV	anti-HBs	HBs-Ag-NT
Serum	19	19	19	19	19	19	19	9
Plasma	6	11	11	11	11	12	12	6
Total	25	30	30	30	30	31	31	15

* Because of limited sample volume, not all spiking tests could be performed for all samples (in this case, only the unspiked sample was analysed and test value quotient of pre- and post-mortem samples was determined).

**Table 2:** Agreement of qualitative pre- and post-mortem serological test results of the examined assays

Assay	Agreement of qualitative pre- and post-mortem test results*
HBsAg	91.2% (62/68) (81.8–96.7% 95% CI)
anti-HBc	98.4% (63/64) (91.6–100% 95% CI)
anti-HBs	96.2% (51/53) (87–99.5% 95% CI)
anti-HCV	100% (60/60) (95.1–100% 95% CI)
anti-HIV	100% (62/62) (95.3–100% 95% CI)
anti-HTLV 1/2	100% (61/61) (95.2–100% 95% CI)
anti-*T. pallidum*	100% (66/66) (95.6–100% 95% CI)

* Irrespective of spiking and used matrix (serum or plasma).

**Table 3:** Distribution of post-/pre-mortem serological test result ratios for each examined assay

PEI	Samples with post-/pre-mortem test result ratio, %	Distribution of ratios in each range, %																				
		HBsAg			anti-HBc			anti-HBs			anti-HCV			anti-HIV			anti-HTLV 1/2			anti-*T. pallidum*		
		un-spiked	spiked		un-spiked	spiked		un-spikedspiked			un-spiked	spiked		un-spikedspiked			un-spiked	spiked		un-spiked	spiked	
			low	high		low	high		low	high		low	high		low	high		low	high		low	high
✓	75–125	46.7	**84.2**	**84.2**	40	**76.5**	**100**	41.9*	**72.7**	**63.6**	43.3	**100**	**100**	30	**81.3**	**100**	64.5	**66.7**	**66.7**	66.7	**94.4**	**94.4**
		(14/30)	(16/19)	(16/19)	(12/30)	(13/17)	(17/17)	(13/31)	(8/11)	(7/11)	(13/30)	(15/15)	(15/15)	(9/30)	(13/16)	(16/16)	(20/31)	(10/15)	(10/15)	(20/30)	(17/18)	(17/18)
✘	>125	3.3	**15.8**	**15.8**	60	–	–	48.4	**27.3**	**18.2**	57.7	–	–	23.3	**12.5**	–	6.5	**33.3**	**26.7**	30	**5.6**	**5.6**
		(1/30)	(3/19)	(3/19)	(18/30)	(0/17)	(0/17)	(15/31)	(3/11)	(2/11)	(17/30)	(0/15)	(0/15)	(7/30)	(2/16)	(0/16)	(2/31)	(5/15)	(4/15)	(9/30)	(1/18)	(1/18)
✘	<75	50	–	–	–	**23.5**	–	9.7	–	**18.2**	–	–	–	46.7	**6.3**	–	29	–	**6.7**	3.3	–	–
		(15/30)	(0/19)	(0/19)	(0/30)	(4/17)	(0/17)	(3/31)	(0/11)	(2/11)	(0/30)	(0/15)	(0/15)	(14/30)	(1/16)	(0/16)	(9/31)	(0/15)	(1/15)	(1/30)	(0/18)	(0/18)

According to PEI requirements, test results of the paired samples should not differ more than 25% for positive-spiked samples (percentages in bold). ✓/✘ = Fulfilling PEI requirements (within/out of the PEI specifications for positive-spiked samples), L, low spike level, H, high spike level.

* Completely pre- and post-mortem negative test results included.

**Table 4:** Summarized HBV-, HCV-, and HIV-PCR sample and QC result characteristics on the examined platforms: all materials (serum and plasma), pre- and post-mortem samples

	Cobas 6800 (Roche)		Alinity m (Abbott)		CAP/CTM (Roche)	
	IU/mL	QC (Ct-value)	IU/mL	QC (Ct-value)	IU/mL	QC (Ct-value)
*HBV*						
Mean	51	34.45	208	27.01	57	28.53
MIN	38	33.54	94	26.25	20	28.00
MAX	78	35.49	532	27.83	155	56.00
SD	9	0.47	89	0.36	33	0.46
CV, %	5	0.16	185	0.10	19	0.13
Five-fold factor range	10–255	–	42–1,040	–	11–285	–

	IU/mL	QC (Ct-value)	IU/mL	QC (Ct-value)	IU/mL	QC (Ct-value)

*HCV*						
Mean	289	36.41	189	21.04	338	32.12
MIN	183	35.72	71	20.42	172	31.60
MAX	427	37.12	295	22.70	549	32.80
SD	50.97	0.30	56.86	0.47	94.10	0.28
CV, %	147.21	0.11	107.27	0.10	318.32	0.09
Five-fold factor range	58–1,445	–	38–945	–	68–1,690	–

	copies/mL	QC (Ct-value)	copies/mL	QC (Ct-value)	copies/mL	QC (Ct-value)

*HIV*						
Mean	145	37.27	143	17.17	165	27.19
MIN	55	36.85	72	16.43	72	26.50
MAX	224	38.06	245	18.66	344	28.00
SD	38	0.30	40	0.66	58	0.32
CV, %	55	0.11	58	0.11	97	0.09
Five-fold factor range	29–725	–	29–715	–	33–825	–
All samples diluted 1:5 prior testing. SD, standard deviation; CV %, coefficient of variation.		
