# Supplementary material for: Suitability of Different Diagnostic Platforms for Virological Testing of Blood Samples from Cornea Donors
Source: Transfus Med Hemother. 2022 Jun 14;49(6):379–87. doi: 10.1159/000524250 (PMC9768292; doi:10.1159/000524250)
Supplement: Supplementary file 1 — Supplementary data [file tmh-0049-0379-s01.docx]

Supplementary Material

- Detailed analysis of the serological parameters test results
- **Table S1** – Serological sample characterisation
- **Table S2** – NAT sample characterisation
- **Table S3** – Test results for the serological parameters **HBsAg, anti-HBc, anti-HCV and anti-HIV**
- **Table S4** – Test results for the serological parameters **anti-*T. pallidum***, **anti-HTLV 1/2** and **anti-HBs**
- **Table S5** – Results of the **HBsAg confirmatory test**
- **Table S6a** – **HBV-DNA** test result summary
- **Table S6b** – *Detailed analysis* of **HBV-DNA** test results
- **Table S7a** – **HCV-RNA** test result summary
- **Table S7b** – *Detailed analysis* of **HCV-RNA** test results
- **Table S8a** – **HIV-RNA** test result summary
- **Table S8b** – *Detailed analysis* of **HIV-RNA** test results
- **Table S9** – Detailed evaluation of internal QC values in the testing for HBV (24 negative post-mortem blood samples)
- **Table S10** – Detailed evaluation of internal QC values in the testing HCV RNA (24 negative post-mortem blood samples)
- **Table S11** – Detailed evaluation of internal QC values in the testing for HIV RNA (24 negative post-mortem blood samples)

**Detailed analysis of the serological parameters test results**

**Analysis of HBV parameters**

*HBsAg*

In the qualitative evaluation, a high agreement of 91.2% for the results of unspiked as well as spiked samples was achieved. In 6 out of 68 analyses (8.82%), a negative result was detected in the pre-mortem serum sample, while the post-mortem sample was found to be weakly reactive (S/CO between 1.45 and 4.59), which could not be verified in the confirmation test (neutralisation test). Ratios of the S/CO values of pre- and post-mortem blood samples ranged between 0.09 and 2.2 for all measurements (i.e. for un-spiked as well as "low" and "high" spiked samples). For 7 measurements (10.3%), the ratio was >1.25, for 46 it was between 0.75 and 1.25 (67.6%) and for 15 measurements (22.1%) it was <0.75. The majority of deviations were observed in the S/CO quotients of unspiked samples for 53.3% (16/30) of conducted analyses in this group.

*HBsAg confirmatory test*

All spiked pre- and post-mortem samples were successfully detected. The degree of neutralisation ranged between 86 - 100%. For unspiked samples and with the exception of one sample, an S/CO value was always generated in the original sample (without addition of the neutralisation reagent) resulting in a negative result in the confirmatory test. For one sample (sample no. 2 - see Tab. S5) the confirmatory test generated an unspecific test result.

*Anti-HBc*

An almost perfect agreement of qualitative results (98.4%) was achieved for both unspiked and spiked pre- and post-mortem samples. Only in 1 of 64 analyses (1.56%) a weakly reactive result (S/CO = 1.78) was detected in the pre-mortem serum sample, while the post-mortem sample proved to be negative (S/CO = 0.74). The ratios of the S/CO values of pre- and post-mortem blood samples ranged between 0.40 and 5.8 for all measurements (i.e. for unspiked as well as "low" and "high" spiked samples). For 18 measurements (28.1%) the value was >1.25, for 42 between 0.75 and 1.25 (65.6%) and for 4 measurements <0.75 (6.3%). The majority of deviations were observed in the S/CO quotients of unspiked samples for 60% (18/30) of conducted analyses in this group.

*Anti-HBs*

A high agreement of 96.2% for qualitative results was achieved for both unspiked and spiked pre- and post-mortem samples. Only in 2 (sample 15) of 53 analyses (3.8%) a weakly reactive result (mIU/mL = 12.58 and 11.86, respectively) was detected in the pre-mortem taken serum and EDTA plasma samples, while the samples taken post-mortem were tested negative with results below the cut-off of 10mIU/mL (mIU/mL = 8.19 and 2.85), respectively.

The ratios of the mIU/mL values of pre- and post-mortem blood samples ranged from 0.30 to 4.6 across all measurements (i.e. for un-spiked as well as for "low" and "high" spiked samples). Only one sample (no. 16 - see table S4) showed significantly higher ratios between pre- and post-mortem samples (15.4 and 20, respectively). The anti-HBs value of the pre-mortem taken serum sample was 230 mIU/mL and of the post-mortem sample 11.5 mIU/mL. This was observed in parallel in both the serum and the EDTA plasma sample (175.05 vs. 11.34 mIU/mL). The same applies to sample no. 9 (see Tab. S4) with mIU/mL values of the pre-mortem (serum and EDTA plasma, respectively) sample of 122.31 and 78.37 and of the post-mortem (serum and EDTA plasma, respectively) samples of 26.56 and 24.06 mIU/mL. In 20 measurements (37.7%), the quotient was >1.25, in 28 between 0.75 - 1.25 (52.8%) and in 5 measurements <0.75 (9.4%). The majority of deviations were observed in the quotients of unspiked samples for 58.1% (18/31) of conducted analyses in this group.

**Analysis of anti-HCV**

A 100% agreement of qualitative results was observed for both unspiked and spiked pre- and post-mortem samples. The S/CO quotients of pre- and post-mortem blood samples ranged from 0.80 to 4.5 for all measurements (i.e. for unspiked, low and high spiked samples), >1.25 for 17 measurements (28.3%), 0.75 to 1.25 for 43 measurements (71.7%). The greatest deviations were observed in the S/CO quotients of unspiked samples for 57.7% (17/30) of conducted analyses in this group.

**Analysis of anti-HIV**

A 100% agreement of qualitative results was achieved for both unspiked and spiked samples for both pre- and post-mortem donations. The S/CO quotients of pre- and post-mortem blood samples ranged from 0.23 to 4.8 for all measurements (i.e. un-spiked, low and high spiked samples), >1.25 for 9 measurements (14.5%), from 0.75 to 1.25 for 38 measurements (61.3%) and <0.75 for 15 measurements (24.2%). The majority of deviations were observed in the S/CO quotients of unspiked samples for 70% (21/30) of conducted analyses in this group.

**Analysis of anti-HTLV 1/2**

A 100% agreement of qualitative results was achieved for both unspiked and spiked samples for both pre- and post-mortem donations. The S/CO quotients of pre- and post-mortem blood samples ranged from 0.60 to 5.0 for all measurements (i.e. for unspiked, low and high spiked samples), >1.25 for 11 measurements (18%), 0.75 to 1.25 for 40 measurements (65.6%) and <0.75 for 10 measurements (16.4%). The majority of deviations were observed in the S/CO quotients of unspiked samples for 35.5% (11/31) of conducted analyses in this group.

**Analysis of anti-*T. pallidum***

A 100% agreement of qualitative results was achieved for both unspiked and spiked samples for both pre- and post-mortem donations. The S/CO quotients of pre- and post-mortem blood samples ranged from 0.60 to 7.0 for all measurements (i.e. for unspiked as well as low and high spiked samples), >1.25 for 11 measurements (16.7%), 0.75 to 1.25 for 54 measurements (81.2%) and <0.75 for 1 measurement (1.5%). The majority of deviations were observed in the S/CO quotients of unspiked and low level spiked samples for 33.3% (10/30 and 5/15) of conducted analyses in this group.

**Table S1** – Serological sample characterisation: Post-mortem samples were collected between 17.58 - 23.83 hours post-mortem. Where available, both pre- and post-mortem serum and EDTA plasma samples were obtained.

| **Individual Nr.** | **PS** | **LS** | **LB**  **EP** | **LS**  **EP** | **BC**  **PM [h]** | **BC PM**  **to -30°C [h:min]** |
| --- | --- | --- | --- | --- | --- | --- |
| 1 | 0 | 1 | 0 | 0 | -54.83 |  |
|  | 1 | 0 | 1 | 0 | 17.92 | 17:55 |
| 2 | 0 | 1 | 0 | 1 | -48.75 |  |
|  | 1 | 0 | 1 | 0 | 23.83 | 23:50 |
| 3 | 0 | 1 | 0 | 1 | -75.12 |  |
|  | 1 | 0 | 1 | 0 | 23.38 | 23:23 |
| 4 | 0 | 1 | 0 | 1 | -34.17 |  |
|  | 1 | 0 | 1 | 0 | 19.08 | 19:05 |
| 5 | 0 | 1 | 0 | 1 | -84.67 |  |
|  | 1 | 0 | 1 | 0 | 17.58 | 17:35 |
| 6 | 0 | 1 | 0 | 1 | -10.42 |  |
|  | 1 | 0 | 1 | 0 | 22.25 | 22:15 |
| 7 | 0 | 1 | 0 | 1 | -29.70 |  |
|  | 1 | 0 | 1 | 0 | 22.97 | 22:58 |
| 8 | 0 | 1 | 0 | 1 | -14.58 |  |
|  | 1 | 0 | 1 | 0 | 21.17 | 21:10 |
| 9 | 0 | 1 | 0 | 1 | -30.82 |  |
|  | 1 | 0 | 1 | 0 | 21.60 | 21:36 |
| 10 | 0 | 1 | 0 | 1 | -80.30 |  |
|  | 1 | 0 | 1 | 0 | 23.53 | 23:32 |
| 11 | 0 | 1 | 0 | 1 | -28.25 |  |
|  | 1 | 0 | 1 | 0 | 21.92 | 21:55 |
| 12 | 0 | 1 | 0 | 1 | -33.20 |  |
|  | 1 | 0 | 1 | 0 | 20.80 | 20:48 |
| 13 | 0 | 1 | 0 | 1 | -11.60 |  |
|  | 1 | 0 | 1 | 0 | 20.15 | 20:09 |
| 14 | 0 | 1 | 0 | 1 | -38.50 |  |
|  | 1 | 0 | 1 | 0 | 21.00 | 21:00 |
| 15 | 0 | 1 | 0 | 1 | -45.63 |  |
|  | 1 | 0 | 1 | 0 | 21.70 | 21:42 |
| 16 | 0 | 1 | 0 | 1 | -49.85 |  |
|  | 1 | 0 | 1 | 0 | 20.15 | 20:09 |
| 17 | 0 | 1 | 0 | 1 | -14.28 |  |
|  | 1 | 0 | 1 | 0 | 20.05 | 20:03 |
| 18 | 0 | 1 | 0 | 1 | -26.08 |  |
|  | 1 | 0 | 1 | 0 | 20.42 | 20:25 |
| 19 | 0 | 1 | 0 | 1 | -30.08 |  |
|  | 1 | 0 | 1 | 0 | 21.08 | 21:05 |

LS = pre-mortem (living donor) serum sample; PS = post-mortem serum sample; LS-EP = pre-mortem EDTA plasma sample;

PS-EP = post-mortem EDTA plasma sample; BC= blood collection; BC PM [h] = blood collection x hours before ( - ) or after occurrence of death;
BC PM to -30°C [h:min]= total period (in hours:minutes) between post-mortem blood collection and start of sample storage at -30°C

**Table S2** – NAT sample characterisation: Post-mortem samples were collected between 16.18 - 23.33 hours post-mortem. Where available, both pre- and post-mortem serum and EDTA plasma samples were obtained.

| **Individual**  **Nr.** | **PS [mL]** | **LS [mL]** | **PS-EP [mL]** | **LS-EP [mL]** | **BC PM [h]** | | **BC PM**  **to -30°C**  **[h:min]** |
| --- | --- | --- | --- | --- | --- | --- | --- |
| 1 | 0 | 2 | 0 | 1 | -32.75 |  |  |
|  | 3.5 | 0 | 2 | 0 |  | 17.37 | 18:30 |
| 2 | 0 | 1 | 0 | 1.5 | -38.50 |  |  |
|  | 4.5 | 0 | 2 | 0 |  | 20.25 | 21:20 |
| 3 | 0 | 1.5 | 0 | 1 | -127.6 |  |  |
|  | 1.5 | 0 | 1.5 | 0 |  | 23.32 | 23:49 |
| 4 | 0 | 1 | 0 | 0 | -53.75 |  |  |
|  | 1.0 | 0 | 1 | 0 |  | 22.25 | 23:51 |
| 5 | 0 | 1 | 0 | 0 | -38.98 |  |  |
|  | 2.0 | 0 | 1 | 0 |  | 16.18 | 17:16 |
| 6 | 0 | 1.5 | 0 | 0 | -148.00 |  |  |
|  | 1.0 | 0 | 1 | 0 |  | 23.33 | 23:50 |
| 7 | 0 | 2.5 | 0 | 1.5 | -63.20 |  |  |
|  | 4.0 | 0 | 4.0 | 0 |  | 18.3 | 18:53 |
| 8 | 0 | 2 | 0 | 1 | -11.33 |  |  |
|  | 1.0 | 0 | 1 | 0 |  | 22.67 | 23:40 |
| 9 | 0 | 1.0 | 0 | 1.5 | -8.20 |  |  |
|  | 2.0 | 0 | 2.0 | 0 |  | 20.22 | 22:18 |

LS = pre-mortem (living donor) serum sample; PS = post-mortem serum sample; LS-EP = pre-mortem EDTA plasma sample;

PS-EP = post-mortem EDTA plasma sample; BC = blood collection; BC PM [h] = blood collection x hours before ( - ) or after occurrence of death;
BC PM to -30°C [h:min] = total period (in hours:minutes) between post-mortem blood collection and start of sample storage at -30°C

**Table S3** – Test results for the serological parameters **HBsAg, anti-HBc, anti-HCV and anti-HIV** of paired pre- and post-mortem blood samples.

| Individual Nr. |  | **HBsAg** (S/CO) | | | **Anti-HBc** (S/CO) | | | **Anti-HCV** (S/CO) | | | **Anti-HIV** (S/CO) | | |
| --- | --- | --- | --- | --- | --- | --- | --- | --- | --- | --- | --- | --- | --- |
|  | Material | unspiked* | low spike  level* | high spike level* | unspiked* | low spike level* | high spike level* | unspiked* | low spike level* | high spike level* | unspiked* | low spike level* | high spike level* |
| 1 | **LS** | **neg.**(0.46) | -- | -- | **neg.** (0.14) | -- | -- | **neg.** (0.10) | **pos.** (3.20) | **pos.** (16.18) | **neg.** (0.24) | **pos.** (2.66) | **pos.** (418.10) |
|  | **PS** | **pos.**(2.48) | -- | -- | **neg.** (0.05) | -- | -- | **neg.** (0.06) | **pos.** (3.25) | **pos.** (16.55) | **neg.** (0.05) | **pos.** (2.24) | **pos.** (410.71) |
|  | **LS/PS**** | **0.19** | -- | -- | **2.8** | -- | -- | **1.7** | **0.98** | **0.98** | **4.8** | **1.2** | **1.02** |
|  | **LS-EP** | -- | -- | -- | -- | -- | -- | -- | -- | -- | -- | -- | -- |
|  | **PS-EP** | -- | -- | -- | -- | -- | -- | -- | -- | -- | -- | -- | -- |
|  | **LS-EP/ PS-EP**** | -- | -- | -- | -- | -- | -- | -- | -- | -- | -- | -- | -- |
| 2 | **LS** | **neg.** (0.41) | -- | -- | **pos.** (1.78) | -- | -- | **neg.** (0.10) | **pos.** (3.52) | **pos.** (17.17) | **neg.** (0.07) | **pos.** (2.96) | **pos.** (418.59) |
|  | **PS** | **pos.** (4.59) | -- | -- | **neg.** (0.74) | -- | -- | **neg**. (0.04) | **pos.** (3.30) | **pos.** (15.91) | **neg.** (0.25) | **pos.** (1.67) | **pos.** (361.23) |
|  | **LS/PS**** | **0.09** | -- | -- | **2.4** | -- | -- | **2.5** | **1.1** | **1.2** | **0.28** | **1.8** | **1.2** |
|  | **LS-EP** | -- | -- | -- | -- | -- | -- | -- | -- | -- | -- | -- | -- |
|  | **PS-EP** | -- | -- | -- | -- | -- | -- | -- | -- | -- | -- | -- | -- |
|  | **LS-EP/ PS-EP**** | -- | -- | -- | -- | -- | -- | -- | -- | -- | -- | -- | -- |
| 3 | **LS** | **neg.** (0.48) | -- | -- | **neg.** (0.21) | -- | -- | **neg.** (0.15) | -- | -- | **neg.** (0.06) | -- | -- |
|  | **PS** | **neg.** (0.34) | -- | -- | **neg.** (0.11) | -- | -- | **neg.** (0.11) | -- | -- | **neg.** (0.06) | -- | -- |
|  | **LS/PS**** | **1.4** | -- | -- | **1.9** | -- | -- | **1.4** | -- | -- | **1.0** | -- | -- |
|  | **LS-EP** | -- | -- | -- | -- | -- | -- | -- | -- | -- | -- | -- | -- |
|  | **PS-EP** | -- | -- | -- | -- | -- | -- | -- | -- | -- | -- | -- | -- |
|  | **LS-EP/ PS-EP**** | -- | -- | -- | -- | -- | -- | -- | -- | -- | -- | -- | -- |
| 4 | **LS** | **neg.** (0.52) | **pos.** (4.47) | **pos.** (37.00) | **neg.**(0.29) | **pos.** (2.14) | **pos.**(8.23) | **neg.**( 0.12) | -- | -- | **neg.** (0.12) | -- | -- |
|  | **PS** | **neg.** (0.60) | **pos.** (4.04) | **pos.** (30.15) | **neg.** (0.28) | **pos.** (2.22) | **pos.** (8.61) | **neg.** (0.10) | -- | -- | **neg.** (0.22) | -- | -- |
|  | **LS/PS**** | **0.9** | **1.1** | **1.23** | **1.04** | **0.96** | **0.96** | **1.2** | -- | -- | **0.55** | -- | -- |
|  | **LS-EP** | -- | -- | -- | -- | -- | -- | -- | -- | -- | -- | -- | -- |
|  | **PS-EP** | -- | -- | -- | -- | -- | -- | -- | -- | -- | -- | -- | -- |
|  | **LS-EP/ PS-EP**** | -- | -- | -- | -- | -- | -- | -- | -- | -- | -- | -- | -- |

**Table S3** (continued) - Test results for the serological parameters **HBsAg, anti-HBc, anti-HCV and anti-HIV** of paired pre- and post-mortem blood samples.

| Individual Nr. |  | **HBsAg** (S/CO) | | | **Anti-HBc** (S/CO) | | | **Anti-HCV** (S/CO) | | | **Anti-HIV** (S/CO) | | |
| --- | --- | --- | --- | --- | --- | --- | --- | --- | --- | --- | --- | --- | --- |
|  | Material | unspiked* | low spike  level* | high spike level* | unspiked* | low spike level* | high spike level* | unspiked* | low spike level* | high spike level* | unspiked* | low spike level* | high spike level* |
| 5 | **LS** | **neg.** (0.35) | **pos.** (3.90) | **pos.** (33.85) | **neg.** (0.08) | **pos.** (1.77) | **pos.** (7.25) | **neg.** (0.07) | **pos.** (3.52) | **pos.** (17.17) | **neg.** (0.08) | **pos.** (2.84) | **pos.** (401.28) |
|  | **PS** | **neg.** (0.44) | **pos.** (4.40) | **pos.** (38.60) | **neg.** (0.06) | **pos.** (3.64) | **pos.** (8.43) | **neg.** (0.05) | **pos.** (3.66) | **pos.** (16.04) | **neg.** (0.05) | **pos.** (2.56) | **pos.** (449.09) |
|  | **LS/PS**** | **0.8** | **0.9** | **0.9** | **1.3** | **0.5** | **0.9** | **1.4** | **0.96** | **1.1** | **1.6** | **1.1** | **0.89** |
|  | **LS-EP** | **neg.** (0.41) | -- | -- | **neg.** (0.08) | -- | -- | **neg.** (0.05) | -- | -- | **neg.** (0.07) | -- | -- |
|  | **PS-EP** | **neg.** (0.48) | -- | -- | **neg.** (0.06) | -- | -- | **neg.** (0.05) | -- | -- | **neg.** (0.05) | -- | -- |
|  | **LS-EP/ PS-EP**** | **0.85** | -- | -- | **1.3** | -- | -- | **1.0** | -- | -- | **1.4** | -- | -- |
| 6 | **LS** | **neg.** (0.45) | -- | -- | **neg.** (0.20) | -- | -- | **neg.** (0.08) | **pos.** (3.67) | **pos.** (15.82) | **neg.** (0.05) | **pos.**(2.31) | **pos.** (419.91) |
|  | **PS** | **neg.** (0.90) | -- | -- | **neg.** (0.08) | -- | -- | **neg.** (0.06) | **pos.** (3.5) | **pos.** (16.23) | **neg.** (0.08) | **pos.** (2.80) | **pos.** (442.88) |
|  | **LS/PS**** | **0.5** | -- | -- | **2.5** | -- | -- | **1.33** | **1.1** | **0.97** | **0.63** | **0.83** | **1.0** |
|  | **LS-EP** | **neg.** (0.23) | **pos.** (4.28) | **pos.** (34.88) | **neg.** (0.12) | **pos.** (2.01) | **pos.** (8.22) | **neg.** (0.07) | -- | -- | **neg.** (0.06) | -- | -- |
|  | **PS-EP** | **neg.** (0.78) | **pos.** (3.24) | **pos.** (28.69) | **neg.** (0.08) | **pos.** (2.10) | **pos.** (8.41) | **neg.** (0.06) | -- | -- | **neg.** (0.06) | -- | -- |
|  | **LS-EP/ PS-EP**** | **0.3** | **1.3** | **1.2** | **1.5** | **1.0** | **1.0** | **1.2** | -- | -- | **1.0** | -- | -- |
| 7 | **LS** | **neg.** (0.42) | **pos.** (4.25) | **pos.** (37.39) | **neg.** (0.18) | **pos.** (1.58) | **pos.** (8.25) | **neg.** (0.10) | **pos.** (3.27) | **pos.** (14.24) | **neg.** (0.06) | **pos.** (2.83) | **pos.** (437.28) |
|  | **PS** | **neg.** (0.40) | **pos.** (3.93) | **pos.** (31.29) | **neg.** (0.21) | **pos.** (1.74) | **pos.** (8.61) | **neg.** (0.10) | **pos.** (3.30) | **pos.** (15.41) | **neg.** (0.08) | **pos** (3.27) | **pos.** (462.39) |
|  | **LS/PS**** | **1.05** | **1.08** | **1.19** | **0.86** | **0.91** | **0.96** | **1.0** | **0.99** | **0.92** | **0.75** | **0.87** | **0.95** |
|  | **LS-EP** | -- | -- | -- | -- | -- | -- | -- | -- | -- | -- | -- | -- |
|  | **PS-EP** | -- | -- | -- | -- | -- | -- | -- | -- | -- | -- | -- | -- |
|  | **LS-EP/ PS-EP**** | -- | -- | -- | -- | -- | -- | -- | -- | -- | -- | -- | -- |
| 8 | **LS** | **neg.** (0.48) | **pos.** (4.72) | **pos.** (40.56) | **neg.** (0.10) | **pos.** (1.42) | **pos.** (8.07) | **neg.** (0.06) | **pos.** (2.94) | **pos.** (15.06) | **neg.** (0.04) | **pos.** (1.97) | **pos.** (363.86) |
|  | **PS** | **neg.** (0.43) | **pos.** (4.47) | **pos.** (39.06) | **neg.** (0.07) | **pos.** (1.63) | **pos.** (8.51) | **neg.** (0.04) | **pos.** (3.29) | **pos.** (16.16) | **neg.** (0.04) | **pos.** (2.51) | **pos.** (415.90) |
|  | **LS/PS**** | **1.1** | **1.1** | **1.04** | **1.43** | **0.87** | **0.95** | **1.5** | **0.89** | **0.93** | **1.0** | **0.79** | **0.88** |
|  | **LS-EP** | -- | -- | -- | -- | -- | -- | -- | -- | -- | -- | -- | -- |
|  | **PS-EP** | -- | -- | -- | -- | -- | -- | -- | -- | -- | -- | -- | -- |
|  | **LS-EP/ PS-EP**** | -- | -- | -- | -- | -- | -- | -- | -- | -- | -- | -- | -- |

**Table S3** (continued) - Test results for the serological parameters **HBsAg, anti-HBc, anti-HCV and anti-HIV** of paired pre- and post-mortem blood samples.

| Individual Nr. |  | **HBsAg** (S/CO) | | | **Anti-HBc** (S/CO) | | | **Anti-HCV** (S/CO) | | | **Anti-HIV** (S/CO) | | |
| --- | --- | --- | --- | --- | --- | --- | --- | --- | --- | --- | --- | --- | --- |
|  | Material | unspiked* | low spike  level* | high spike level* | unspiked* | low spike level* | high spike level* | unspiked* | low spike level* | high spike level* | unspiked* | low spike level* | high spike level* |
| 9 | **LS** | **neg.** (0.35) | **pos.** (3.79) | **pos.** (55.70) | **neg.** (0.24) | **pos.** (1.49) | **pos.** (7.39) | **neg.** (0.17) | **pos.** (3.18) | **pos.** (16.25) | **neg.** (0.08) | **pos.** (3.54) | **pos.** (467.51) |
|  | **PS** | **neg.** (0.59) | **pos.** (4.50) | **pos.** (25.13) | **neg.** (0.23) | **pos.** (2.01) | **pos.** (7.80) | **neg.** (0.07) | **pos.** (3.19) | **pos.** (15.55) | **neg.** (0.11) | **pos.** (2.91) | **pos.** (514.92) |
|  | **LS/PS**** | **0.6** | **0.84** | **2.2** | **1.04** | **0.74** | **0.95** | **2.43** | **1** | **1.1** | **0.72** | **1.2** | **0.91** |
|  | **LS-EP** | **neg.** (0.44) | -- | -- | **neg.** (0.20) | -- | -- | **neg.** (0.12) | -- | -- | **neg.** (0.06) | -- | -- |
|  | **PS-EP** | **neg.** (0.70) | -- | -- | **neg.** (0.22) | -- | -- | **neg.** (0.07) | -- | -- | **neg.** (0.11) | -- | -- |
|  | **LS-EP/ PS-EP**** | **0.63** | -- | -- | **0.91** | -- | -- | **1.7** | -- | -- | **0.6** | -- | -- |
| 10 | **LS** | **neg.** (0.43) | -- | -- | **neg.** (0.09) | -- | -- | **neg.** (0.08) | -- | -- | **neg.** (0.04) | -- | -- |
|  | **PS** | **neg.** (0.37) | -- | -- | **neg.** (0.06) | -- | -- | **neg.** (0.06) | -- | -- | **neg.** (0.06) | -- | -- |
|  | **LS/PS**** | **1.2** | -- | -- | **1.5** | -- | -- | **1.33** | -- | -- | **0.7** | -- | -- |
|  | **LS-EP** | **neg.** (0.41) | -- | -- | **neg.** (0.09) | -- | -- | **neg.** (0.08) | -- | -- | **neg.** (0.05) | -- | -- |
|  | **PS-EP** | **neg.** (0.51) | -- | -- | **neg.** (0.06) | -- | -- | **neg.** (0.06) | -- | -- | **neg.** (0.05) | -- | -- |
|  | **LS-EP/ PS-EP**** | **0.8** | -- | -- | **1.5** | -- | -- | **1.33** | -- | -- | **1** | -- | -- |
| 11 | **LS** | **neg.** (0.38) | **pos.** (4.45) | **pos.** (55.90) | **neg.** (0.12) | **pos.** (2.05) | **pos.** (7.99) | **neg.** (0.06) | -- | -- | **neg.** (0.05) | -- | -- |
|  | **PS** | **pos.** (4.31) | **pos.** (4.04) | **pos.** (36.15) | **neg.** (0.10) | **pos.** (2.11) | **pos.** (7.70) | **neg.** (0.04) | -- | -- | **neg.** (0.11) | -- | -- |
|  | **LS/PS**** | **0.09** | **1.1** | **1.6** | **1.2** | **1** | **1.04** | **1.5** | **--** | **--** | **0.5** | **--** | **--** |
|  | **LS-EP** | **neg.** (0.36) | **pos.** (4.49) | **pos.** (50.19) | **neg.** (0.22) | -- | -- | **neg.** (0.09) | -- | -- | **neg.** (0.06) | -- | -- |
|  | **PS-EP** | **pos.** (1.45) | **pos.** (3.22) | **pos.** (36.59) | **neg.** (0.11) | -- | -- | **neg.** (0.04) | -- | -- | **neg.** (0.12) | -- | -- |
|  | **LS-EP/ PS-EP**** | **0.93** | **1.4** | **1.4** | **2** | -- | -- | **2.3** | -- | -- | **0.5** | -- | -- |
| 12 | **LS** | **neg.** (0.37) | **pos.** (4.05) | **pos.** (32.77) | **neg.** (0.15) | -- | -- | **neg.** (0.16) | **pos.** (3.55) | **pos.** (15.58) | **neg.** (0.05) | **pos.** (2.85) | **pos.** (440.28) |
|  | **PS** | **neg.** (0.50) | **pos.** (4.51) | **pos.** (37.92) | **neg.** (0.09) | -- | -- | **neg.** (0.11) | **pos.** (3.60) | **pos.** (17.26) | **neg.** (0.08) | **pos.** (4.01) | **pos.** (504.93) |
|  | **LS/PS**** | **0.74** | **0.9** | **0.9** | **1.7** | -- | -- | **1.5** | **1** | **0.9** | **0.63** | **0.7** | **0.9** |
|  | **LS-EP** | **neg.** (0.39) | -- | -- | **neg.** (0.08) | **pos.** (1.56) | **pos.** (7.91) | **neg.** (0.11) | -- | -- | **neg.** (0.05) | -- | -- |
|  | **PS-EP** | **neg.** (0.38) | -- | -- | **neg.** (0.07) | **pos.** (1.42) | **pos.** (7.89) | **neg.** (0.10) | -- | -- | **neg.** (0.08) | -- | -- |
|  | **LS-EP/ PS-EP**** | **1.03** | -- | -- | **1.1** | **1.1** | **1** | **1.1** | -- | -- | **0.63** | -- | -- |

**Table S3** (continued) - Test results for the serological parameters **HBsAg, anti-HBc, anti-HCV and anti-HIV** of paired pre- and post-mortem blood samples.

| Individual Nr. |  | **HBsAg** (S/CO) | | | **Anti-HBc** (S/CO) | | | **Anti-HCV** (S/CO) | | | **Anti-HIV** (S/CO) | | |
| --- | --- | --- | --- | --- | --- | --- | --- | --- | --- | --- | --- | --- | --- |
|  | Material | unspiked* | low spike  level* | high spike level* | unspiked* | low spike level* | high spike level* | unspiked* | low spike level* | high spike level* | unspiked* | low spike level* | high spike level* |
| 13 | **LS** | **neg.** (0.45) | **pos.** (4.55) | **pos.** (35.43) | **neg.** (0.08) | **pos.** (1.14) | **pos.** (8.07) | **neg.** (0.13) | **pos.** (2.89) | **pos.** (15.02) | **neg.** (0.05) | **pos.** (2.62) | **pos.** (417.72) |
|  | **PS** | **neg.** (0.63) | **pos.** (4.12) | **pos.** (35.17) | **neg.** (0.05) | **pos.** (1.81) | **pos.** (8.34) | **neg.** (0.10) | **pos.** (3.05) | **pos.** (14.84) | **neg.** (0.06) | **pos.** (2.72) | **pos.** (444.17) |
|  | **LS/PS**** | **0.7** | **1.1** | **1** | **1.6** | **0.6** | **1** | **1.3** | **1** | **1** | **0.87** | **1** | **0.9** |
|  | **LS-EP** | **neg.** (0.37) | **pos.** (4.20) | **pos.** (35.60) | **neg.** (0.06) | **pos.** (1.71) | **pos.** (8.49) | **neg.** (0.10) | **pos.** (3.31) | **pos.** (14.28) | **neg.** (0.13) | **pos.** (2.58) | **pos.** (424.63) |
|  | **PS-EP** | **neg.** (0.53) | **pos.**(2.85) | **pos.** (35.6) | **neg.** (0.06) | **pos.** (1.44) | **pos.** (8.19) | **neg.** (0.09) | **pos.** (3.16) | **pos.** (14.78) | **neg.** (0.09) | **pos.** (2.32) | **pos.** (428.38) |
|  | **LS-EP/ PS-EP**** | **0.7** | **1.5** | **1** | **1** | **1.2** | **1** | **1.1** | **1.1** | **1** | **1.4** | **1.1** | **1** |
| 14 | **LS** | **neg.** (0.35) | **pos.** (4.91) | **pos.** (43.36) | **neg.** (0.13) | **pos.** (1.55) | **pos.** (8.23) | **neg.** (0.06) | **pos.** (3.46) | **pos.** (15.41) | **neg.** (0.07) | **pos.** (2.60) | **pos.** (417.70) |
|  | **PS** | **neg.** (0.86) | **pos.** (4.71) | **pos.** (38.69) | **neg.** (0.06) | **pos.** (1.63) | **pos.** (8.60) | **neg.** (0.04) | **pos.** (3.47) | **pos.** (15.28) | **neg.** (0.18) | **pos.** (2.59) | **pos.** (473.66) |
|  | **LS/PS**** | **0.41** | **1** | **1.1** | **2.2** | **1** | **1** | **1.5** | **1** | **1** | **0.4** | **1** | **0.9** |
|  | **LS-EP** | -- | -- | -- | -- | -- | -- | -- | -- | -- | -- | -- | -- |
|  | **PS-EP** | -- | -- | -- | -- | -- | -- | -- | -- | -- | -- | -- | -- |
|  | **LS-EP/ PS-EP**** | -- | -- | -- | -- | -- | -- | -- | -- | -- | -- | -- | -- |
| 15 | **LS** | **neg.** (0.34) | **pos.** (5.06) | **pos.** (37.52) | **neg.** (0.11) | **pos.** (1.49) | **pos.** (8.11) | **neg.** (0.07) | **pos.** (3.65) | **pos.** (17.42) | **neg.** (0.10) | **pos.** (2.22) | **pos.** (388.25) |
|  | **PS** | **neg.** (0.36) | **pos.** (4.13) | **pos.** (34.81) | **neg.** (0.08) | **pos.** (1.63) | **pos.** (8.13) | **neg.** (0.06) | **pos.** (3.34) | **pos.** (15.93) | **neg.** (0.05) | **pos.** (2.94) | **pos.** (431.13) |
|  | **LS/PS**** | **0.9** | **1.2** | **1.1** | **1.4** | **0.9** | **1** | **1.2** | **1.1** | **1.1** | **2** | **0.8** | **0.9** |
|  | **LS-EP** | **neg.** (0.35) | **pos.** (4.61) | **pos.** (39.45) | **neg.** (0.10) | **pos.** (1.55) | **pos.** (8.00) | **neg.** (0.06) | **pos.** (3.43) | **pos.** (17.09) | **neg.** (0.10) | **pos.** (2.42) | **pos.** (397.88) |
|  | **PS-EP** | **neg.** (0.41) | **pos.** (4.28) | **pos.** (36.79) | **neg.** (0.09) | **pos.** (1.43) | **pos**. (8.04) | **neg.** (0.06) | **pos.** (3.26) | **pos.** (16.84) | **neg.** (0.06) | **pos.** (3.24) | **pos.** (458.19) |
|  | **LS-EP/ PS-EP**** | **0.9** | **1.1** | **1.1** | **1.1** | **1.1** | **1** | **1** | **1.1** | **1** | **1.7** | **0.8** | **0.9** |
| 16 | **LS** | **neg.** (0.47) | **pos.** (3.74) | **pos.** (30.37) | **pos.** (7.26) | **pos.** (1.49) | **pos.** (8.11) | **neg.** (0.09) | -- | -- | **neg.** (0.06) | **pos.** (2.94) | **pos.** (400.41) |
|  | **PS** | **neg.** (0.93) | **pos.** (4.91) | **pos.** (35.64) | **pos.** (1.26) | **pos.** (1.63) | **pos.** (8.13) | **neg.** (0.02) | -- | -- | **neg.** (0.08) | **pos.** (3.14) | **pos.** (504.76) |
|  | **LS/PS**** | **0.5** | **0.8** | **0.9** | **5.8** | **0.9** | **1** | **4.5** | -- | -- | **0.8** | **0.9** | **0.8** |
|  | **LS-EP** | **neg.** (0.45) | -- | -- | **pos.** (7.04) | **pos.** (1.43) | **pos.** (8.04) | **neg.** (0.07) | -- | -- | **neg.** (0.07) | -- | -- |
|  | **PS-EP** | **neg.** (0.79) | -- | -- | **pos.** (1.29) | **pos.** (1.55) | **pos.** (8.0) | **neg.** (0.02) | -- | -- | **neg.** (0.10) | -- | -- |
|  | **LS-EP/ PS-EP**** | **0.6** | -- | -- | **5.5** | **0.9** | **1** | **3.5** | -- | -- | **0.7** | -- | -- |

**Table S3** (continued) - Test results for the serological parameters **HBsAg, anti-HBc, anti-HCV and anti-HIV** of paired pre- and post-mortem blood samples.

| Individual Nr. |  | **HBsAg** (S/CO) | | | **Anti-HBc** (S/CO) | | | **Anti-HCV** (S/CO) | | | **Anti-HIV** (S/CO) | | |
| --- | --- | --- | --- | --- | --- | --- | --- | --- | --- | --- | --- | --- | --- |
|  | Material | unspiked* | low spike  level* | high spike level* | unspiked* | low spike level* | high spike level* | unspiked* | low spike level* | high spike level* | unspiked* | low spike level* | high spike level* |
| 17 | **LS** | **neg.** (0.49) | **pos.** (4.79) | **pos.** (40.45) | **neg.** (0.12) | -- | -- | **neg.** (0.08) | -- | -- | **neg.** (0.07) | -- | -- |
|  | **PS** | **neg.** (0.64) | **pos.** (4.77) | **pos.** (40.70) | **neg.** (0.11) | -- | -- | **neg.** (0.07) | -- | -- | **neg.** (0.05) | -- | -- |
|  | **LS/PS**** | **0.8** | **1** | **1** | **1.1** | -- | -- | **1.1** | -- | -- | **1.4** | -- | -- |
|  | **LS-EP** | **neg.** (0.52) | **pos.** (4.81) | **pos.** (36.71) | **neg.** (0.12) | -- | -- | **neg.** (0.06) | -- | -- | **neg.** (0.10) | -- | -- |
|  | **PS-EP** | **neg.** (0.51) | **pos.** (4.86) | **pos.** (39.94) | **neg.** (0.12) | -- | -- | **neg.** (0.07) | -- | -- | **neg.** (0.09) | -- | -- |
|  | **LS-EP/ PS-EP**** | **1** | **1** | **0.9** | **1** | -- | -- | **0.9** | -- | -- | **1.1** | -- | -- |
| 18 | **LS** | **neg.** (0.34) | **pos.** (4.99) | **pos.** (37.43) | **neg.** (0.15) | **pos.**(1.68) | **pos.** (7.62) | **neg.** (0.12) | **pos.** (2.85) | **pos.** (15.04) | **neg.** (0.03) | **pos.** (2.28) | **pos.** (418.38) |
|  | **PS** | **neg.** (0.44) | **pos.** (4.18) | **pos.** (32.78) | **neg.** (0.11) | **pos.** (4.78) | **pos.** (8.24) | **neg.** (0.10) | **pos.** (3.51) | **pos.** (15.11) | **neg.** (0.13) | **pos.** (2.46) | **pos.** (476.80) |
|  | **LS/PS**** | **0.8** | **1.2** | **1.1** | **1.4** | **0.4** | **0.9** | **1.2** | **0.8** | **1** | **0.23** | **0.9** | **0.9** |
|  | **LS-EP** | -- | -- | -- | -- | -- | -- | -- | -- | -- | -- | -- | -- |
|  | **PS-EP** | -- | -- | -- | -- | -- | -- | -- | -- | -- | -- | -- | -- |
|  | **LS-EP/ PS-EP**** | -- | -- | -- | -- | -- | -- | -- | -- | -- | -- | -- | -- |
| 19 | **LS** | **neg.** (0.36) | **pos.** (4.54) | **pos.** (40.05) | **neg.** (0.18) | **pos.** (1.64) | **pos.** (8.65) | **neg.** (0.14) | **pos.** (3.45) | **pos.** (15.74) | **neg.** (0.12) | **pos.** (2.41) | **pos.** (409.65) |
|  | **PS** | **pos.** (3.54) | **pos.** (5.14) | **pos.** (33.49) | **neg.** (0.17) | **pos.** (1.59) | **pos.** (8.36) | **neg.** (0.14) | **pos.** (3.76) | **pos.** (16.84) | **neg.** (0.10) | **pos.** (1.78) | **pos.** (371.64) |
|  | **LS/PS**** | **0.1** | **0.9** | **1.2** | **1.1** | **1.03** | **1.04** | **1** | **0.92** | **0.94** | **1.2** | **1.4** | **1.1** |
|  | **LS-EP** | **neg.** (0.48) | -- | -- | **neg.** (0.18) | -- | -- | **neg.** (0.13) | -- | -- | **neg.** (0.09) | -- | -- |
|  | **PS-EP** | **pos.** (2.07) | -- | -- | **neg.** (0.21) | -- | -- | **neg.** (0.13) | -- | -- | **neg.** (0.20) | -- | -- |
|  | **LS-EP/ PS-EP**** | **0.23** | -- | -- | **0.9** | -- | -- | **1** | -- | -- | **0.5** | -- | -- |

*qualitative test result and corresponding S/CO-value; **S/CO-value ratio of pre- and post-mortem sample; neg. = negative; pos. = positive; LS = pre-mortem (living donor) serum sample;

PS = post-mortem serum sample; LS-EP = pre-mortem EDTA plasma sample; PS-EP = post-mortem EDTA plasma sample; -- = not analysed (not enough volume)

**Table S4** - Test results for the serological parameters **anti-*T. pallidum***, **anti-HTLV** **1/2** and **anti-HBs** of paired pre- and post-mortem blood samples.

| Individual Nr. | Material | **Anti-*T. pallidum*** (S/CO) | | | **Anti-HTLV 1/2** (S/CO) | | | **Anti-HBs** (mIU/mL) | | |
| --- | --- | --- | --- | --- | --- | --- | --- | --- | --- | --- |
|  |  | unspiked* | low spike  level* | high spike  level* | unspiked* | low spike  level* | high spike  level* | unspiked*** | low spike  level*** | high spike  level*** |
| 1 | **LS** | **neg.** (0.09) | **pos.** (2.97) | **pos.** (9.50) | **neg.** (0.09) | **pos.** (3.97) | **pos.** (89.90) | **neg.** (0.21) | **pos.** (14.96) | **pos.** (14.96) |
|  | **PS** | **neg.** (0.09) | **pos.** (2.84) | **pos.** (9.38) | **neg.** (0.16) | **pos.** (1.87) | **pos.** (55.62) | **neg.** (0.72) | **pos.** (14.07) | **pos.** (53.59) |
|  | **LS/PS**** | **1** | **1.1** | **1.01** | **0.6** | **2.1** | **1.6** | **0.3** | **1.1** | **0.3** |
|  | **LS-EP** | -- | -- | -- | -- | -- | -- | -- | -- | -- |
|  | **PS-EP** | -- | -- | -- | -- | -- | -- | -- | -- | -- |
|  | **LS-EP/ PS-EP**** | -- | -- | -- | -- | -- | -- | -- | -- | -- |
| 2 | **LS** | **neg.** (0.03) | **pos.** (2.80) | **pos.** (9.05) | **neg.** (0.17) | **pos.** (3.08) | **pos.** (78.06) | **pos.** (>1000) | -- | -- |
|  | **PS** | **neg.** (0.05) | **pos.** (2.54) | **pos.** (9.72) | **neg.** (0.33) | **pos.** (1.97) | **pos.** (46.34) | **pos.** (896.53) | -- | -- |
|  | **LS/PS**** | **0.6** | **1.1** | **0.9** | **0.5** | **1.6** | **1.7** | **1.1** | -- | -- |
|  | **LS-EP** | -- | -- | -- | -- | -- | -- | -- | -- | -- |
|  | **PS-EP** | -- | -- | -- | -- | -- | -- | -- | -- | -- |
|  | **LS-EP/ PS-EP**** | -- | -- | -- | -- | -- | -- | -- | -- | -- |
| 3 | **LS** | **neg.** (0.12) | -- | -- | **neg.** (0.10) | -- | -- | **neg.** (0.00) | **pos.** (15.65) | **pos.** (53.07) |
|  | **PS** | **neg.** (0.09) | -- | -- | **neg.** (0.12) | -- | -- | **neg.** (0.00) | **pos.** (16.42) | **pos.** (55.79) |
|  | **LS/PS**** | **1.3** | -- | -- | **0.8** | -- | -- |  | **0.95** | **0.95** |
|  | **LS-EP** | -- | -- | -- | -- | -- | -- | -- | -- | -- |
|  | **PS-EP** | -- | -- | -- | -- | -- | -- | -- | -- | -- |
|  | **LS-EP/ PS-EP**** | -- | -- | -- | -- | -- | -- | -- | -- | -- |
| 4 | **LS** | **neg.** (0.11) | -- | -- | **neg.** (0.12) | -- | -- | **neg.** (0.00) | -- | -- |
|  | **PS** | **neg.** (0.09) | -- | -- | **neg.** (0.13) | -- | -- | **neg.** (0.22) | -- | -- |
|  | **LS/PS**** | **1.2** | -- | -- | **0.9** | -- | -- |  | -- | -- |
|  | **LS-EP** | -- | -- | -- | -- | -- | -- | -- | -- | -- |
|  | **PS-EP** | -- | -- | -- | -- | -- | -- | -- | -- | -- |
|  | **LS-EP/ PS-EP**** | -- | -- | -- | -- | -- | -- | -- | -- | -- |

**Table S4** (continued) - Test results for the serological parameters **anti-*T. pallidum***, **anti-HTLV 1/2** and **anti-HBs** of paired pre- and post-mortem blood samples.

| Individual Nr. | Material | **Anti-*T. pallidum*** (S/CO) | | | **Anti-HTLV 1/2** (S/CO) | | | **Anti-HBs** (mIU/mL) | | |
| --- | --- | --- | --- | --- | --- | --- | --- | --- | --- | --- |
|  |  | unspiked* | low spike  level* | high spike  level* | unspiked* | low spike  level* | high spike  level* | unspiked*** | low spike  level*** | high spike  level*** |
| 5 | **LS** | **neg.** (0.06) | **pos.** (3.45) | **pos.** (10.60) | **neg.** (0.10) | -- | -- | **neg.** (0.00) | **pos.** (14.59) | **pos.** ( 47.27) |
|  | **PS** | **neg.** (0.06) | **pos.** (3.17) | **pos.** (10.94) | **neg.** (0.14) | -- | -- | **neg.** (0.00) | **pos.** (17.39) | **pos.** (60.43) |
|  | **LS/PS**** | **1** | **1.1** | **1** | **0.7** | -- | -- |  | **0.84** | **0.7** |
|  | **LS-EP** | **neg.** (0.05) | -- | -- | **neg.** (0.13) | -- | -- | **neg.** (0.00) | -- | -- |
|  | **PS-EP** | **neg.** (0.05) | -- | -- | **neg.** (0.22) | -- | -- | **neg.** (0.00) | -- | -- |
|  | **LS-EP/ PS-EP**** | **1** | -- | -- | **0.6** | -- | -- |  | -- | -- |
| 6 | **LS** | **neg.** (0.12) | -- | -- | **neg.** (0.11) | -- | -- | **pos.** (46.43) | -- | -- |
|  | **PS** | **neg.** (0.08) | -- | -- | **neg.** (0.15) | -- | -- | **pos.** (65.46) | -- | -- |
|  | **LS/PS**** | **1.5** | **--** | **--** | **0.73** | **--** | **--** | **0.71** | **--** | **--** |
|  | **LS-EP** | **neg.** (0.08) | **pos.** (3.22) | **pos.** (10.32) | **neg.** (0.11) | **pos.** (3.94) | **pos.** (94.69) | **pos.** (44.86) | **pos.** (60.62) | **pos.** (90.50) |
|  | **PS-EP** | **neg.** (0.08) | **pos.** (3.23) | **pos.** (10.75) | **neg.** (0.15) | **pos.** (2.59) | **pos.** (76.71) | **pos.** (26.44) | **pos.** (79.43) | **pos.** (110.37) |
|  | **LS-EP/ PS-EP**** | **1** | **1** | **1** | **0.73** | **1.5** | **1.2** | **1.7** | **0.8** | **0.82** |
| 7 | **LS** | **neg.** (0.05) | **pos.** (2.78) | **pos.** (9.53) | **neg.** (0.11) | **pos.** (3.51) | **pos.** (86.24) | **neg.** (0.98) | -- | -- |
|  | **PS** | **neg.** (0.05) | **pos.** (3.34) | **pos.** (9.99) | **neg.** (0.10) | **pos.** (4.24) | **pos.** (96.81) | **neg.** (2.16) | -- | -- |
|  | **LS/PS**** | **1** | **0.83** | **1** | **1.1** | **0.83** | **0.9** | **0.5** | **--** | **--** |
|  | **LS-EP** | -- | -- | -- | -- | -- | -- | -- | -- | -- |
|  | **PS-EP** | -- | -- | -- | -- | -- | -- | -- | -- | -- |
|  | **LS-EP/ PS-EP**** | -- | -- | -- | -- | -- | -- | -- | -- | -- |
| 8 | **LS** | **neg.** (0.04 | **pos.** (2.46) | **pos.** (8.48) | **neg.** (0.06) | **pos.** (3.76) | **pos.** (72.38) | **neg.** (0.10) | **pos.** (16.05) | **pos.** (53.72) |
|  | **PS** | **neg.** (0.04 | **pos.** (2.95) | **pos.** (10.36) | **neg.** (0.07) | **pos.** (4.13) | **pos.** (98.77) | **neg.** (0.00) | **pos.** (17.33) | **pos.** (59.06) |
|  | **LS/PS**** | **1** | **0.8** | **0.82** | **0.9** | **0.9** | **0.73** |  | **0.93** | **0.91** |
|  | **LS-EP** | -- | -- | -- | -- | -- | -- | -- | -- | -- |
|  | **PS-EP** | -- | -- | -- | -- | -- | -- | -- | -- | -- |
|  | **LS-EP/ PS-EP**** | -- | -- | -- | -- | -- | -- | -- | -- | -- |

**Table S4** (continued) - Test results for the serological parameters **anti-*T. pallidum***, **anti-HTLV 1/2** and **anti-HBs** of paired pre- and post-mortem blood samples.

| Individual Nr. | Material | **Anti-*T. pallidum*** (S/CO) | | | **Anti-HTLV 1/2** (S/CO) | | | **Anti-HBs** (mIU/mL) | | |
| --- | --- | --- | --- | --- | --- | --- | --- | --- | --- | --- |
|  |  | unspiked* | low spike  level* | high spike  level* | unspiked* | low spike  level* | high spike  level* | unspiked*** | low spike  level*** | high spike  level*** |
| 9 | **LS** | **neg.** (0.07) | **pos.** (3.02) | **pos.** (9.67) | **neg.** (0.13) | -- | -- | **pos.** (122.31) | -- | -- |
|  | **PS** | **neg.** (0.05) | **pos.** (2.55) | **pos.** (10.21) | **neg.** (0.16) | -- | -- | **pos.** (26.56) | -- | -- |
|  | **LS/PS**** | **1.4** | **1.2** | **1** | **0.81** | **--** | **--** | **4.6** | **--** | **--** |
|  | **LS-EP** | **neg.** (0.05) | -- | -- | **neg.** (0.13) | -- | -- | **pos.** (78.37) | -- | -- |
|  | **PS-EP** | **neg.** (0.04) | -- | -- | **neg.** (0.16) | -- | -- | **pos.** (24.06) | -- | -- |
|  | **LS-EP/ PS-EP**** | **1.25** | **--** | **--** | **0.81** | **--** | **--** | **3.3** | **--** | **--** |
| 10 | **LS** | **neg.** (0.06) | **pos.** (3.25) | **pos.** (9.43) | **neg.** (0.11) | **pos.** (3.79) | **pos.** (93.69) | **neg.** (0.00) | **pos.** (23.06) | **pos.** (55.78) |
|  | **PS** | **neg.** (0.05) | **pos.** (3.36) | **pos.** (9.89) | **neg.** (0.11) | **pos.** (3.97) | **pos.** (97.02) | **neg.** (0.00) | **pos.** (16.72) | **pos.** (45.97) |
|  | **LS/PS**** | **1.2** | **1** | **1** | **1** | **1** | **1** |  | **1.4** | **1.2** |
|  | **LS-EP** | **neg.** (0.06) | **pos.** (3.28) | **pos.** (10.09) | **neg.** (0.11) | **pos.** (5.12) | **pos.** (102.22) | **neg.** (0.00) | -- | -- |
|  | **PS-EP** | **neg.** (0.05) | **pos.** (3.42) | **pos.** (10.45) | **neg.** (0.11) | **pos.** (3.18) | **pos.** (104.01) | **neg.** (0.00) | -- | -- |
|  | **LS-EP/ PS-EP**** | **1.2** | **1** | **1** | **1** | **1.6** | **1** |  | **--** | **--** |
| 11 | **LS** | **neg.** (0.04) | -- | -- | **neg.** (0.11) | -- | -- | **neg.** (6.20) | -- | -- |
|  | **PS** | **neg.** (0.03) | -- | -- | **neg.** (0.14) | -- | -- | **neg.** (2.19) | -- | -- |
|  | **LS/PS**** | **1.3** | **--** | **--** | **0.8** | **--** | **--** | **2.8** | **--** | **--** |
|  | **LS-EP** | **neg.** (0.07) | -- | -- | **neg.** (0.10) | -- | -- | **neg.** (5.07) | -- | -- |
|  | **PS-EP** | **neg.** (0.04) | -- | -- | **neg.** (0.15) | -- | -- | **neg.** (1.92) | -- | -- |
|  | **LS-EP/ PS-EP**** | **1.75** | **--** | **--** | **0.7** | **--** | **--** | **2.6** | **--** | **--** |
| 12 | **LS** | **neg.** (0.07) | -- | -- | **neg.** (0.11) | -- | -- | **neg.** (3.78) | -- | -- |
|  | **PS** | **neg.** (0.05) | -- | -- | **neg.** (0.12) | -- | -- | **neg.** (3.58) | -- | -- |
|  | **LS/PS**** | **1.4** | **--** | **--** | **0.92** | **--** | **--** | **1.1** | **--** | **--** |
|  | **LS-EP** | **neg.** (0.06) | **pos.** (2.67) | **pos.** (9.67) | **neg.** (0.13) | **pos.** (4.20) | **pos.** (94.35) | **neg.** (3.68) | -- | -- |
|  | **PS-EP** | **neg.** (0.07) | **pos.** (2.90) | **pos.** (9.33) | **neg.** (0.12) | **pos.** (3.54) | **pos.** (87.85) | **neg.** (2.27) | -- | -- |
|  | **LS-EP/ PS-EP**** | **0.9** | **0.92** | **1.04** | **1.1** | **1.2** | **1.1** | **1.6** | **--** | **--** |

**Table S4** (continued) - Test results for the serological parameters **anti-*T. pallidum***, **anti-HTLV 1/2** and **anti-HBs** of paired pre- and post-mortem blood samples.

| Individual Nr. | Material | **Anti-*T. pallidum*** (S/CO) | | | **Anti-HTLV 1/2** (S/CO) | | | **Anti-HBs** (mIU/mL) | | |
| --- | --- | --- | --- | --- | --- | --- | --- | --- | --- | --- |
|  |  | unspiked* | low spike  level* | high spike  level* | unspiked* | low spike  level* | high spike  level* | unspiked*** | low spike  level*** | high spike  level*** |
| 13 | **LS** | **neg.** (0.12) | **pos.** (3.01) | **pos.** (9.53) | **neg.** (0.10) | **pos.** (3.85) | **pos.** (94.17) | **pos.** (57.06) | -- | -- |
|  | **PS** | **neg.** (0.11) | **pos.** (3.49) | **pos.** (11.78) | **neg.** (0.09) | **pos.** (3.46) | **pos.** (85.84) | **pos.** (38.34) | -- | -- |
|  | **LS/PS**** | **1.1** | **0.9** | **0.8** | **1.1** | **1.1** | **1.1** | **1.5** | **--** | **--** |
|  | **LS-EP** | **neg.** (0.11) | **pos.** (3.04) | **pos.** (10.63) | **neg.** (0.10) | **pos.** (3.98) | **pos.** (85.36) | **pos.** (67.26) | -- | -- |
|  | **PS-EP** | **neg.** (0.12) | **pos.** (3.52) | **pos.** (10.96) | **neg.** (0.11) | **pos.** (3.81) | **pos.** (80.73) | **pos.** (32.56) | -- | -- |
|  | **LS-EP/ PS-EP**** | **0.92** | **0.9** | **1** | **0.91** | **1.1** | **1.1** | **2.1** | **--** | **--** |
| 14 | **LS** | **neg.** (0.06) | **pos.** (2.67) | **pos.** (9.0) | **neg.** (0.09) | -- | -- | **pos.** (817.09) | -- | -- |
|  | **PS** | **neg.** (0.05) | **pos.** (2.96) | **pos.** (10.03) | **neg.** (0.11) | -- | -- | **pos.** (516.39) | -- | -- |
|  | **LS/PS**** | **1.2** | **0.9** | **0.9** | **0.8** | **--** | **--** | **1.6** | **--** | **--** |
|  | **LS-EP** | -- | -- | -- | **neg.** (0.09) | **pos.** (3.33) | **pos.** (83.87) | **pos.** (>1000) | **pos.** (> 1000) | **pos.** (>1000) |
|  | **PS-EP** | -- | -- | -- | **neg.** (0.11) | **pos.** (3.05) | **pos.** (80.49) | **pos.** (526.28) | **pos.** (532.49) | **pos.** (508.23) |
|  | **LS-EP/ PS-EP**** | -- | -- | -- | **0.82** | **1.1** | **1.04** | **1.9** | **1.9** | **2** |
| 15 | **LS** | **neg.** (0.04) | **pos.** (2.65) | **pos.** (9.98) | **neg.** (0.07) | **pos.** (4.45) | **pos.** (94.68) | **pos.** (12.58) | **pos.** (27.46) | **pos.** (60.89) |
|  | **PS** | **neg.** (0.03) | **pos.** (2.74) | **pos.** (9.47) | **neg.** (0.10) | **pos.** (5.08) | **pos.** (97.13) | **neg.** (8.19) | **pos.** (24.93) | **pos.** (57.59) |
|  | **LS/PS**** | **1.3** | **1** | **1.1** | **0.7** | **0.9** | **1** | **1.5** | **1.1** | **1.1** |
|  | **LS-EP** | **neg.** (0.03) | **pos.** (2.57) | **pos.** (9.29) | **neg.** (0.08) | -- | -- | **pos.** (11.86) | **pos.** (24.98) | **pos.** (58.70) |
|  | **PS-EP** | **neg.** (0.03) | **pos.** (2.97) | **pos.** (9.94) | **neg.** (0.08) | -- | -- | **neg.** (2.85) | **pos.** (7.29) | **pos.** (13.68) |
|  | **LS-EP/ PS-EP**** | **1** | **0.9** | **0.9** | **1** | **--** | **--** | **4.2** | **3.4** | **4.3** |
| 16 | **LS** | **neg.** (0.07) | **pos.** (2.75) | **pos.** (10.61) | **neg.** (0.10) | -- | -- | **pos.** (230.05) | -- | -- |
|  | **PS** | **neg.** (0.01) | **pos.** (1.46) | **pos.** (7.93) | **neg.** (0.02) | -- | -- | **pos.** (11.5) | -- | -- |
|  | **LS/PS**** | **7** | **1.9** | **1.3** | **5** | **--** | **--** | **20** | **--** | **--** |
|  | **LS-EP** | **neg.** (0.06) | -- | -- | **neg.** (0.10) | **pos.** (3.89) | **pos.** (89.40) | **pos.** (175.05) | -- | -- |
|  | **PS-EP** | **neg.** (0.01) | -- | -- | **neg.** (0.13) | **pos.** (4.14) | **pos.** (88.67) | **pos.** (11.34) | -- | -- |
|  | **LS-EP/ PS-EP**** | **6** | **--** | **--** | **0.8** | **0.94** | **1** | **15.4** | **--** | **--** |

**Table S4** (continued) - Test results for the serological parameters **anti-*T. pallidum***, **anti-HTLV 1/2** and **anti-HBs** of paired pre- and post-mortem blood samples.

| Individual Nr. | Material | **Anti-*T. pallidum*** (S/CO) | | | **Anti-HTLV 1/2** (S/CO) | | | **Anti-HBs** (mIU/mL) | | |
| --- | --- | --- | --- | --- | --- | --- | --- | --- | --- | --- |
|  |  | unspiked* | low spike  level* | high spike  level* | unspiked* | low spike  level* | high spike  level* | unspiked*** | low spike  level*** | high spike  level*** |
| 17 | **LS** | **neg.** (0.08) | -- | -- | **neg.** (0.11) | -- | -- | **neg.** (0.75) | -- | -- |
|  | **PS** | **neg.** (0.08) | -- | -- | **neg.** (0.10) | -- | -- | **neg.** (0.29) | -- | -- |
|  | **LS/PS**** | **1** | **--** | **--** | **1.1** | **--** | **--** | **2.6** | **--** | **--** |
|  | **LS-EP** | **neg.** (0.08) | -- | -- | **neg.** (0.11) | -- | -- | **neg.** (0.00) | -- | -- |
|  | **PS-EP** | **neg.** (0.08) | -- | -- | **neg.** (0.08) | -- | -- | **neg.** (0.48) | -- | -- |
|  | **LS-EP/ PS-EP**** | **1** | **--** | **--** | **1.4** | **--** | **--** |  | **--** | **--** |
| 18 | **LS** | **neg.** (0.07) | **pos.** (3.50) | **pos.** (11.09) | **neg.** (0.10) | **pos.** (6.41) | **pos.** (93.24) | **neg.** (0.28) | **pos.** (15.34) | **pos.** (51.05) |
|  | **PS** | **neg.** (0.06) | **pos.** (3.14) | **pos.** (10.37) | **neg.** (0.10) | **pos.** (5.76) | **pos.** (74.48) | **neg.** (0.37) | **pos.** (18.20) | **pos.** (59.11) |
|  | **LS/PS**** | **1.2** | **1.1** | **1.1** | **1** | **1.1** | **1.3** | **0.8** | **0.84** | **0.9** |
|  | **LS-EP** | -- | -- | -- | -- | -- | -- | -- | -- | -- |
|  | **PS-EP** | -- | -- | -- | -- | -- | -- | -- | -- | -- |
|  | **LS-EP/ PS-EP**** | -- | -- | -- | -- | -- | -- | -- | -- | -- |
| 19 | **LS** | **neg.** (0.10) | **pos.** (3.23) | **pos.** (10.56) | **neg.** (0.09) | **pos.** (4.05) | **pos.** (97.66) | **neg.** (0.00) | **pos.** (16.04) | **pos.** (53.75) |
|  | **PS** | **neg.** (0.08) | **pos.** (3.07) | **pos.** (10.2) | **neg.** (0.08) | **pos.** (2.25) | **pos.** (46.74) | **neg.** (0.00) | **pos.** (15.54) | **pos.** (51.58) |
|  | **LS/PS**** | **1.25** | **1.1** | **1** | **1.1** | **1.8** | **2.1** |  | **1** | **1.04** |
|  | **LS-EP** | **neg.** (0.09) | -- | -- | **neg.** (0.09) | -- | -- | **neg.** (0.00) | -- | -- |
|  | **PS-EP** | **neg.** (0.08) | -- | -- | **neg.** (0.13) | -- | -- | **neg.** (0.21) | -- | -- |
|  | **LS-EP/ PS-EP**** | **1.13** | **--** | **--** | **0.7** | **--** | **--** |  | **--** | **--** |

*qualitative test result and corresponding S/CO-value; **S/CO-value or mIU/mL ratio of pre- and post-mortem sample; ***qualitative test result and corresponding mIU/mL;

neg. = negative; pos. = positive; LS = pre-mortem (living donor) serum sample; PS = post-mortem serum sample; LS-EP = pre-mortem EDTA plasma sample;

PS-EP = post-mortem EDTA plasma sample; -- = not analysed (not enough volume)

**Table S5** – Results of the **HBsAg confirmatory test** of paired pre- and post-mortem blood samples.

| Individual Nr. | Material | **original sample**  **HBsAg confirmatory test** | | | **„low spike level“ sample**  **HBsAg confirmatory test** | | | **„high spike level“ sample**  **HBsAg confirmatory test** | | |
| --- | --- | --- | --- | --- | --- | --- | --- | --- | --- | --- |
|  |  | sample with neutralization reagent (qualitative) S/CO C1 | original sample (qualitative)  S/CO  C2 | degree of neutralization  (%) | sample with neutralization reagent (qualitative) S/CO C1 | original sample (qualitative)  S/CO  C2 | degree of neutralization  (%) | sample with neutralization reagent (qualitative) S/CO C1 | original sample (qualitative)  S/CO  C2 | degree of neutralization  (%) |
| 1 | **LS** | -- | -- | -- | -- | -- | -- | -- | -- | -- |
|  | **PS** | (neg.) 0.33 | (neg.) 0.34 | not applicable* | -- | -- | -- | -- | -- | -- |
|  | **LS-EP** | -- | -- | -- | -- | -- | -- | -- | -- | -- |
|  | **PS-EP** | -- | -- | -- | -- | -- | -- | -- | -- | -- |
| 2 | **LS** | -- | -- | -- | -- | -- | -- | -- | -- | -- |
|  | **PS** | (neg.) 0.81 | (pos.) 0.79 | non-specific** | -- | -- | -- | -- | -- | -- |
|  | **LS-EP** | -- | -- | -- | -- | -- | -- | -- | -- | -- |
|  | **PS-EP** | -- | -- | -- | -- | -- | -- | -- | -- | -- |
| 3 | **LS** | -- | -- | -- | -- | -- | -- | -- | -- | -- |
|  | **PS** | -- | -- | -- | -- | -- | -- | -- | -- | -- |
|  | **LS-EP** | -- | -- | -- | -- | -- | -- | -- | -- | -- |
|  | **PS-EP** | (neg.) 0.17 | (neg.) 0.20 | not applicable* | (neg.) 0.19 | (pos.) 4.10 | 100 | (neg.) 0.24 | (pos.) 36.65 | 100 |
| 5 | **LS** | -- | -- | -- | -- | -- | -- | -- | -- | -- |
|  | **PS** | -- | -- | -- | -- | -- | -- | -- | -- | -- |
|  | **LS-EP** | (neg.) 0.19 | (neg.) 0.27 | not applicable* | (neg.) 0.20 | (pos.) 4.99 | 100 | (neg.) 0.23 | (pos.) 38.52 | 100 |
|  | **PS-EP** | (neg.) 0.18 | (neg.) 0.23 | not applicable* | (neg.) 0.25 | (pos.) 5.12 | 99 | -- | -- | -- |
| 6 | **LS** | -- | -- | -- | -- | -- | -- | -- | -- | -- |
|  | **PS** | (neg.) 0.52 | (neg.) 0.53 | not applicable* | (neg.) 0.45 | (pos.) 4.54 | 94 | (neg.) 0.45 | (pos.) 33.44 | 99 |
|  | **LS-EP** | -- | -- | -- | -- | -- | -- | -- | -- | -- |
|  | **PS-EP** | -- | -- | -- | -- | -- | -- | -- | -- | -- |

**Table S5** (continued) – Results of the **HBsAg confirmatory test** of paired pre- and post-mortem blood samples.

| Individual Nr. | | Material | | | **original sample**  **HBsAg confirmatory test** | | | **„low spike level“ sample**  **HBsAg confirmatory test** | | | **„high spike level“ sample**  **HBsAg confirmatory test** | | |  |
| --- | --- | --- | --- | --- | --- | --- | --- | --- | --- | --- | --- | --- | --- | --- |
|  |  |  | | | sample with neutralization reagent (qualitative) S/CO C1 | original sample (qualitative)  S/CO  C2 | degree of neutralization  (%) | sample with neutralization reagent (qualitative) S/CO C1 | original sample (qualitative)  S/CO  C2 | degree of neutralization  (%) | sample with neutralization reagent (qualitative) S/CO C1 | original sample (qualitative)  S/CO  C2 | degree of neutralization  (%) |  |
| 8 | | **LS** | | | (neg.) 0.25 | (neg.) 0.22 | not applicable* | (neg.) 0.22 | (pos.) 5.54 | 100 | (neg.) 0.20 | (pos.) 45.16 | 100 |  |
|  |  | **PS** | | | -- | -- | -- | (neg.) 3824 | (pos.) 5.18 | 96 | (neg.) 0.24 | (pos.) 43.91 | 100 |  |
|  |  | **LS-EP** | | | -- | -- | -- | -- | -- | -- | -- | -- | -- |  |
|  |  | **PS-EP** | | | -- | -- | -- | -- | -- | -- | -- | -- | -- |  |
| 10 | **LS** | | | -- | | -- | -- | -- | -- | -- | -- | -- | -- |  |
|  | **PS** | | | (neg.) 0.22 | | (neg.) 0.16 | not applicable* | (neg.) 0.21 | (pos.) 5.04 | 100 | (neg.) 0.18 | (pos.) 41.50 | 100 |  |
|  | **LS-EP** | | | -- | | -- | -- | -- | -- | -- | -- | -- | -- |  |
|  | **PS-EP** | | | -- | | -- | -- | -- | -- | -- | -- | -- | -- |  |
| 11 | | **LS** | | |  |  |  |  |  |  |  |  |  |  |
|  |  | **PS** | | | (neg.) 0.63 | (pos.) 0.74 | non-specific** (19) | (neg.) 0.74 | (pos.) 34.82 | 98 | (neg.) 0.67 | (pos.) 4.36 | 89 |  |
|  |  | **LS-EP** | | | -- | -- | -- | -- | -- | -- | -- | -- | -- |  |
|  |  | **PS-EP** | | | (neg.) 0.87 | (pos.) 1.15 | non-specific**  (30) | (neg.) 0.92 | (pos.) 35.03 | 98 | (neg.) 0.88 | (pos.) 5.07 | 86 |  |
| 12 | | **LS** | | -- | | | -- | -- | -- | -- | -- | -- | -- | -- |
|  |  | **PS** | | (neg.) 0.46 | | | (neg.) 0.24 | not applicable* | (neg.) 0.22 | (pos.) 5.63 | 100 | (neg.) 0.22 | (pos.) 42.13 | 100 |
|  |  | **LS-EP** | | -- | | | -- | -- | -- | -- | -- | -- | -- | -- |
|  |  | **PS-EP** | | -- | | | -- | -- | -- | -- | -- | -- | -- | -- |
| 13 | | | **LS** | | | (neg.) 0.21 | (neg.) 0.22 | not applicable* | (neg.) 0.27 | (pos.) 4.94 | 102 | (pos.) 1.51 | (pos.) 41.02 | 97 |
|  |  |  | **PS** | | | (neg.) 0.27 | (neg.) 0.26 | not applicable* | (neg.) 0.27 | (pos.) 4.76 | 98 | (neg.) 0.27 | (pos.) 37.68 | 100 |
|  |  |  | **LS-EP** | | | -- | -- | -- | -- | -- | -- | -- | -- | -- |
|  |  |  | **PS-EP** | | | -- | -- | -- | -- | -- | -- | -- | -- | -- |

**Table S5** (continued) – Results of the **HBsAg confirmatory test** of paired pre- and post-mortem blood samples.

| Individual Nr. | Material | | | **original sample**  **HBsAg confirmatory test** | | | | **„low spike level“ sample**  **HBsAg confirmatory test** | | | **„high spike level“ sample**  **HBsAg confirmatory test** | | |
| --- | --- | --- | --- | --- | --- | --- | --- | --- | --- | --- | --- | --- | --- |
|  |  | | | sample with neutralization reagent (qualitative) S/CO C1 | | original sample (qualitative)  S/CO  C2 | degree of neutralization  (%) | sample with neutralization reagent (qualitative) S/CO C1 | original sample (qualitative)  S/CO  C2 | degree of neutralization  (%) | sample with neutralization reagent (qualitative) S/CO C1 | original sample (qualitative)  S/CO  C2 | degree of neutralization  (%) |
| 14 | **LS** | | -- | | | -- | -- | -- | -- | -- | -- | -- | -- |
|  | **PS** | | -- | | | -- | -- | -- | -- | -- | -- | -- | -- |
|  | **LS-EP** | | (neg.) 0.22 | | | (neg.) 0.23 | not applicable* | (neg.) 0.17 | (pos.) 1.39 | 102 | (neg.) 0.23 | (pos.) 10.13 | 100 |
|  | **PS-EP** | | -- | | | -- | -- | -- | -- | -- | -- | -- | -- |
| 16 | | **LS** | | | -- | -- | -- | -- | -- | -- | -- | -- | -- |
|  |  | **PS** | | | -- | -- | -- | -- | -- | -- | -- | -- | -- |
|  |  | **LS-EP** | | | -- | -- | -- | -- | -- | -- | -- | -- | -- |
|  |  | **PS-EP** | | | (neg.) 0.24 | (neg.) 0.26 | not applicable* | (neg.) 0.25 | (pos.) 5.52 | 99 | (neg.) 0.30 | (pos.) 40.86 | 100 |
| 17 | | **LS** | | -- | | -- | -- | -- | -- | -- | -- | -- | -- |
|  |  | **PS** | | (neg.) 0.27 | | (neg.) 0.29 | not applicable* | (neg.) 0.28 | (pos.) 5.42 | 98 | (neg.) 0.27 | (pos.) 43.10 | 100 |
|  |  | **LS-EP** | | -- | | -- | -- | -- | -- | -- | -- | -- | -- |
|  |  | **PS-EP** | | -- | | -- | -- | -- | -- | -- | -- | -- | -- |
| 19 | | **LS** | | -- | | -- | -- | -- | -- | -- | -- | -- | -- |
|  |  | **PS** | | -- | | -- | -- | -- | -- | -- | -- | -- | -- |
|  |  | **LS-EP** | | (neg.) 0.17 | | (neg.) 0.19 | not applicable* | (neg.) 0.22 | (pos.) 4.93 | 100 | -- | -- | -- |
|  |  | **PS-EP** | | (neg.) 0.35 | | (neg.) 0.49 | not applicable* | (neg.) 0.41 | (pos.) 4.55 | 95 | (neg.) 0.35 | (pos.) 31.69 | 100 |

*since the S/CO-value of the original sample without and with neutralisation (NT) reagent yielded a negative result (S/CO value < 1), a degree of neutralisation could not be calculated - in routine diagnostics, the original sample would be determined as "non-specific" and the result of the confirmatory assay as "negative"; **the S/CO-value of the C1-sample (including NT reagent) did not neutralize >50% (degree of neutralization < 50%) - In routine diagnostics, the original sample would be determined as "non-specific" and the result of the confirmatory assay as "negative"; neg. = negative; pos. = positive; LS = pre-mortem (living donor) serum sample; PS = post-mortem serum sample; LS-EP = pre-mortem EDTA plasma sample; PS-EP = post-mortem EDTA plasma sample; -- = not analysed (not enough volume)

**Table S6a** – **HBV-DNA** test result summary of spiked paired pre- and post-mortem blood samples.

| Individual Nr. | All samples are diluted 1:5 | **Cobas 6800**  (Roche) | | | **Alinity m**  (Abbott) | | | **Cobas Ampliprep/Cobas TaqMan** (Roche) | | |
| --- | --- | --- | --- | --- | --- | --- | --- | --- | --- | --- |
|  |  | Result  (IU/mL) | Result  (Ct-value) | QC  (Ct-value) | Result  (IU/mL) | Result  (Ct-value) | QC  (Ct-value) | Result  (IU/mL) | Result  (Ct-value) | QC  (Ct-value) |
| 1 | **PS** | 38 | 35.23 | 34.1 | 119 | 30.35 | 27.37 | 115 | 34.4 | 28.9 |
|  | **LS** | 54 | 34.93 | 34.34 | 111 | 30.45 | 27.39 | 49 | 35 | 28.5 |
|  | **PS-EP** | 41 | 36.09 | 35.08 | 168 | 29.84 | 26.71 | invalid | -- | -- |
|  | **LS-EP** | 41 | 36.27 | 35.26 | 142 | 30.09 | 26.87 | 75 | 34.4 | 28.4 |
| 2 | **PS** | 44 | 35.08 | 34.18 | 300 | 29.51 | 26.25 | 50 | 34.8 | 28.2 |
|  | **LS** | 48 | 35.39 | 34.61 | 94 | 30.69 | 27.39 | 39 | 35.4 | 28.5 |
|  | **PS-EP** | 60 | 35.13 | 34.7 | 220 | 29.45 | 26.51 | 53 | 34.7 | 28.2 |
|  | **LS-EP** | 41 | 35.85 | 34.85 | 148 | 30.03 | 26.86 | 84 | 34.3 | 28.4 |
| 3 | **PS** | 55 | 35.14 | 34.57 | 231 | 29.38 | 26.71 | 41 | 36 | 29.2 |
|  | **LS** | 70 | 34.57 | 34.35 | 116 | 30.38 | 27.28 | 20 | 37.4 | 28.3 |
|  | **PS-EP** | 56 | 34.9 | 34.35 | 286 | 29.07 | 26.41 | 37 | 35.1 | 28.1 |
|  | **LS-EP** | 46 | 35.37 | 34.53 | 131 | 30.2 | 27.37 | 155 | 34.8 | 29.8 |
| 4 | **PS** | 49 | 34.29 | 33.57 | invalid | -- | -- | invalid | -- | -- |
|  | **LS** | 52 | 35.13 | 34.47 | 209 | 29.53 | 26.75 | 39 | 35 | 28.2 |
|  | **PS-EP** | 50 | 35.24 | 34.54 | 164 | 29.88 | 26.83 | 37 | 35.3 | 28.3 |
|  | **LS-EP** | 44 | 35.43 | 34.56 | 155 | 29.96 | 27.02 | 47 | 35.4 | 28.7 |
| 5* | **PS** | 40 | 34.78 | 33.73 | 189 | 29.67 | 27.24 | 20 | 37.1 | 29.1 |
|  | **LS** | 66 | 34.48 | 34.19 | 166 | 29.86 | 27.05 | 79 | 34.1 | 28.1 |
| 6* | **PS** | 53 | 34.86 | 34.25 | 144 | 30.07 | 26.95 | 57 | 34.4 | 28 |
|  | **LS** | 57 | 35.44 | 34.93 | 149 | 30.02 | 27.16 | 36 | 35.3 | 28.3 |
| 7 | **PS** | 54 | 35.04 | 34.57 | 256 | 29.75 | 26.85 | invalid | -- | -- |
|  | **LS** | 44 | 35.09 | 34.13 | 241 | 29.84 | 27.32 | n.a. | -- | -- |
|  | **PS-EP** | 42 | 34.44 | 33.54 | 249 | 29.79 | 27.22 | n.a. | -- | -- |
|  | **LS-EP** | 58 | 34.58 | 34.01 | 174 | 30.33 | 27.11 | n.a. | -- | -- |

**Table S6a** (continued) – **HBV-DNA** test result summary of spiked paired pre- and post-mortem blood samples.

| Individual Nr. | All samples are diluted 1:5 | **Cobas 6800**  (Roche) | | | **Alinity m**  (Abbott) | | | **Cobas Ampliprep/Cobas TaqMan** (Roche) | | |
| --- | --- | --- | --- | --- | --- | --- | --- | --- | --- | --- |
|  |  | Result  (IU/mL) | Result  (Ct-value) | QC  (Ct-value) | Result  (IU/mL) | Result  (Ct-value) | QC  (Ct-value) | Result  (IU/mL) | Result  (Ct-value) | QC  (Ct-value) |
| 8 | **PS** | 51 | 34.76 | 34.1 | 314 | 29.44 | 26.72 | n.a. | -- | -- |
|  | **LS** | 56 | 35.44 | 35.44 | 107 | 31.07 | 27.83 | n.a. | -- | -- |
|  | **PS-EP** | 53 | 35.49 | 35.49 | 532 | 28.64 | 26.29 | n.a. | -- | -- |
|  | **LS-EP** | 50 | 35.82 | 34.11 | 232 | 29.9 | 26.94 | n.a. | -- | -- |
| 9 | **PS** | 56 | 34.76 | 34.23 | 254 | 29.76 | 27.4 | n.a. | -- | -- |
|  | **LS** | 57 | 34.94 | 34.43 | 236 | 29.87 | 27.08 | n.a. | -- | -- |
|  | **PS-EP** | 56 | 35.03 | 34.51 | 363 | 29.22 | 26.96 | n.a. | -- | -- |
|  | **LS-EP** | 41 | 35.21 | 34.21 | 256 | 29.7 | 27.34 | n.a. | -- | -- |
| QC | neg. Plasma (dilution plasma) | negative | -- | 35.35 | negative | -1 | 27.24 | negative | -- | 28.8 |
|  | spiked 1:5 diluted neg. plasma | 78 | 35.1 | 35.05 | 199 | 30.13 | 27.28 | 50 | 35.5 | 28.9 |

QC = internal quality control; All samples were diluted 1:5 with negative plasma prior spiking; LS = pre-mortem (living donor) serum sample; PS = post-mortem serum sample; LS-EP = pre-mortem EDTA plasma sample; PS-EP = post-mortem EDTA plasma sample; *not enough volume of EDTA plasma for analysis; n.a. = not analysed (not enough volume); invalid = not evaluable (cause unknown); n.e. = not evaluable

**Table S6b** – Detailed analysis of **HBV-DNA** test results of spiked paired pre- and post-mortem blood samples.

| Individual  Nr. | All samples are diluted 1:5 | **Cobas 6800**  (Roche) | | | **Alinity m**  (Abbott) | | | **Cobas Ampliprep/Cobas TaqMan**  (Roche) | | |
| --- | --- | --- | --- | --- | --- | --- | --- | --- | --- | --- |
|  |  | Result  (IU/mL) | Result  (Ct-value) | QC  (Ct-value) | Result  (IU/mL) | Result  (Ct-value) | QC  (Ct-value) | Result  (IU/mL) | Result  (Ct-value) | QC  (Ct-value) |
| 1 | **PS** | 38 | 35.2 | 34.1 | 119 | 30.4 | 27.4 | 115 | 34.4 | 28.9 |
|  | **LS** | 54 | 34.9 | 34.3 | 111 | 30.5 | 27.4 | 49 | 35.0 | 28.5 |
|  | **Mean** | 46 | 35.1 | 34.2 | 115 | 30.4 | 27.4 | 82 | 34.7 | 28.7 |
|  | **SD** | 11.7 | 0.2 | 0.2 | 5.7 | 0.1 | 0.0 | 46.5 | 0.4 | 0.3 |
|  |  |  |  |  |  |  |  |  |  |  |
| 1 | **PS-EP** | 41 | 36.1 | 35.1 | 168 | 29.8 | 26.7 | invalid | -- | -- |
|  | **LS-EP** | 41 | 36.3 | 35.3 | 142 | 30.1 | 26.9 | 75 | 34.4 | 28.4 |
|  | **Mean** | 41 | 36.2 | 35.2 | 155 | 30.0 | 26.8 | 75 | 34.4 | 28.4 |
|  | **SD** | 0.2 | 0.1 | 0.1 | 18.4 | 0.2 | 0.1 | n.e. | n.e. | n.e. |
|  |  |  |  |  |  |  |  |  |  |  |
| 2 | **PS** | 44 | 35.1 | 34.2 | 300 | 29.5 | 26.3 | 50 | 34.8 | 28.2 |
|  | **LS** | 48 | 35.4 | 34.6 | 94 | 30.7 | 27.4 | 39 | 35.4 | 28.5 |
|  | **Mean** | 46 | 35.2 | 34.4 | 197 | 30.1 | 26.8 | 45 | 35.1 | 28.4 |
|  | **SD** | 2.8 | 0.2 | 0.3 | 146 | 0.8 | 0.8 | 8.1 | 0.4 | 0.2 |
|  | | | | | | | | | | |
| 2 | **PS-EP** | 60 | 35.1 | 34.7 | 220 | 29.5 | 26.5 | 53 | 34.7 | 28.2 |
|  | **LS-EP** | 41 | 35.9 | 34.9 | 148 | 30.0 | 26.9 | 84 | 34.3 | 28.4 |
|  | **Mean** | 51 | 35.5 | 34.8 | 184 | 29.7 | 26.7 | 68 | 34.5 | 28.3 |
|  | **SD** | 13.6 | 0.5 | 0.1 | 50.9 | 0.4 | 0.2 | 21.4 | 0.3 | 0.1 |

| 3 | **PS** | 55 | 35.1 | 34.6 | 231 | 29.4 | 26.7 | 41 | 36.0 | 29.2 |
| --- | --- | --- | --- | --- | --- | --- | --- | --- | --- | --- |
|  | **LS** | 70 | 34.6 | 34.4 | 116 | 30.4 | 27.3 | 20 | 37.4 | 28.3 |
|  | ***Mean*** | 62 | 34.9 | 34.5 | 174 | 29.9 | 27.0 | 31 | 36.7 | 28.8 |
|  | ***SD*** | 10.4 | 0.4 | 0.2 | 81.3 | 0.7 | 0.4 | 15.1 | 1.0 | 0.6 |
|  |  |  |  |  |  |  |  |  |  |  |
| 3 | **PS-EP** | 56 | 34.9 | 34.4 | 286 | 29.1 | 26.4 | 37 | 35.1 | 28.1 |
|  | **LS-EP** | 46 | 35.4 | 34.5 | 131 | 30.2 | 27.4 | 155 | 34.8 | 29.8 |
|  | ***Mean*** | 51 | 35.1 | 34.4 | 209 | 29.6 | 26.9 | 96 | 35.0 | 29.0 |
|  | ***SD*** | 6.9 | 0.3 | 0.1 | 109.6 | 0.8 | 0.7 | 83.7 | 0.2 | 1.2 |

**Table S6b** (continued) – Detailed analysis of **HBV-DNA** test results of spiked paired pre- and post-mortem blood samples.

| Individual Nr. | All samples are diluted 1:5 | **Cobas 6800**  (Roche) | | | **Alinity m**  (Abbott) | | | **Cobas Ampliprep/Cobas TaqMan**  (Roche) | | |
| --- | --- | --- | --- | --- | --- | --- | --- | --- | --- | --- |
|  |  | Result  (IU/mL) | Result  (Ct-value) | QC  (Ct-value) | Result  (IU/mL) | Result  (Ct-value) | QC  (Ct-value) | Result  (IU/mL) | Result  (Ct-value) | QC  (Ct-value) |
| 4 | **PS** | 49 | 34.3 | 33.6 | invalid | -- | -- | invalid | -- | -- |
|  | **LS** | 52 | 35.1 | 34.5 | 209 | 29.5 | 26.8 | 39 | 35.0 | 28.2 |
|  | **Mean** | 50 | 34.7 | 34.0 | 209 | 29.5 | 26.8 | 39 | 36.0 | 14.1 |
|  | **SD** | 1.6 | 0.6 | 0.6 | n.e. | n.e. | n.e. | n.e. | n.e. | n.e. |
|  |  |  |  |  |  |  |  |  |  |  |
| 4 | **PS-EP** | 50 | 35.2 | 34.5 | 164 | 29.9 | 26.8 | 37 | 35.3 | 28.3 |
|  | **LS-EP** | 44 | 35.4 | 34.6 | 155 | 30.0 | 27.0 | 47 | 35.4 | 28.7 |
|  | **Mean** | 47 | 35.3 | 34.6 | 160 | 29.9 | 26.9 | 42 | 35.4 | 28.5 |
|  | **SD** | 4.2 | 0.1 | 0.0 | 6.4 | 0.1 | 0.1 | 6.7 | 0.1 | 0.3 |

| 5* | **PS** | 40 | 34.8 | 33.7 | 189 | 29.7 | 27.2 | 20 | 37.1 | | 29.1 |
| --- | --- | --- | --- | --- | --- | --- | --- | --- | --- | --- | --- |
|  | **LS** | 66 | 34.5 | 34.2 | 166 | 29.9 | 27.1 | 79 | 34.1 | | 28.1 |
|  | **Mean** | 53 | 34.6 | 34.0 | 178 | 29.8 | 27.1 | 50 | 35.6 | | 28.6 |
|  | **SD** | 18.7 | 0.2 | 0.3 | 16.3 | 0.1 | 0.1 | 41.7 | 2.1 | | 0.7 |
|  |  |  |  |  |  |  |  |  | |  |  |
| 6* | **PS** | 53 | 34.9 | 34.3 | 144 | 30.1 | 27.0 | 57 | | 34.4 | 28.0 |
|  | **LS** | 57 | 35.4 | 34.9 | 149 | 30.0 | 27.2 | 36 | | 35.3 | 28.3 |
|  | **Mean** | 55 | 35.2 | 34.6 | 147 | 30.0 | 27.1 | 46 | | 34.9 | 28.2 |
|  | **SD** | 2.5 | 0.4 | 0.5 | 3.5 | 0.0 | 0.1 | 14.9 | | 0.6 | 0.2 |
|  |  |  |  |  |  |  |  |  | |  |  |
| 7 | **PS** | 54 | 35.0 | 34.6 | 256 | 29.8 | 26.9 | invalid | | -- | -- |
|  | **LS** | 44 | 35.1 | 34.1 | 241 | 29.8 | 27.3 | invalid | | -- | -- |
|  | **Mean** | 49 | 35.1 | 34.4 | 249 | 29.8 | 27.1 | n.e. | | n.e. | n.e. |
|  | **SD** | 7.1 | 0.0 | 0.3 | 10.6 | 0.1 | 0.3 | n.e. | | n.e. | n.e. |
|  |  |  |  |  |  |  |  |  | |  |  |
| 7 | **PS-EP** | 42 | 34.4 | 33.5 | 249 | 29.8 | 27.2 | invalid | | -- | -- |
|  | **LS-EP** | 58 | 34.6 | 34.0 | 174 | 30.3 | 27.1 | invalid | | -- | -- |
|  | **Mean** | 50 | 34.5 | 33.8 | 212 | 30.1 | 27.2 | n.e. | | n.e. | n.e. |
|  | **SD** | 11.3 | 0.1 | 0.3 | 53.0 | 0.4 | 0.1 | n.e. | | n.e. | n.e. |

**Table S6b** (continued) – Detailed analysis of **HBV-DNA** test results of spiked paired pre- and post-mortem blood samples.

| Individual Nr. | All samples are diluted 1:5 | **Cobas 6800**  **(Fa. Roche)** | | | **Alinity m**  **(Fa. Abbott)** | | | **Cobas Ampliprep/Cobas TaqMan**  **(Fa. Roche)** | | |
| --- | --- | --- | --- | --- | --- | --- | --- | --- | --- | --- |
|  |  | Result  (IU/mL) | Result  (Ct-value) | QC  (Ct-value) | Result  (IU/mL) | Result  (Ct-value) | QC  (Ct-value) | Result  (IU/mL) | Result  (Ct-value) | QC  (Ct-value) |
| 8 | **PS** | 51 | 34.8 | 34.1 | 314 | 29.4 | 26.7 | invalid | -- | -- |
|  | **LS** | 56 | 35.4 | 35.4 | 107 | 31.1 | 27.8 | invalid | -- | -- |
|  | **Mean** | 54 | 35.1 | 34.8 | 211 | 30.3 | 27.3 | n.e. | n.e. | n.e. |
|  | **SD** | 3.5 | 0.5 | 0.9 | 146.4 | 1.2 | 0.8 | n.e. | n.e. | n.e. |
|  |  |  |  |  |  |  |  |  |  |  |
| 8 | **PS-EP** | 53 | 35.5 | 35.5 | 532 | 28.6 | 26.3 | invalid | -- | -- |
|  | **LS-EP** | 50 | 35.8 | 34.1 | 232 | 29.9 | 26.9 | invalid | -- | -- |
|  | **Mean** | 52 | 35.7 | 34.8 | 382 | 29.3 | 26.6 | n.e. | n.e. | n.e. |
|  | **SD** | 2.1 | 0.2 | 1.0 | 212.1 | 0.9 | 0.5 | n.e. | n.e. | n.e. |

| 9 | **PS** | 56 | 34.8 | 34.2 | 254 | 29.8 | 27.4 | n.a. | -- | -- |
| --- | --- | --- | --- | --- | --- | --- | --- | --- | --- | --- |
|  | **LS** | 57 | 34.9 | 34.4 | 236 | 29.9 | 27.1 | n.a. | -- | -- |
|  | **Mean** | 57 | 34.9 | 34.3 | 245 | 29.8 | 27.2 | n.a. | -- | -- |
|  | **SD** | 0.7 | 0.1 | 0.1 | 12.7 | 0.1 | 0.2 | n.a. | -- | -- |
|  |  |  |  |  |  |  |  |  |  |  |
| 9 | **PS-EP** | 56 | 35.0 | 34.5 | 363 | 29.2 | 27.0 | n.a. | -- | -- |
|  | **LS-EP** | 41 | 35.2 | 34.2 | 256 | 29.7 | 27.3 | n.a. | -- | -- |
|  | **Mean** | 49 | 35.1 | 34.4 | 310 | 29.5 | 27.2 | n.a. | -- | -- |
|  | **SD** | 10.6 | 0.1 | 0.2 | 75.7 | 0.3 | 0.3 | n.a. | -- | -- |

All samples were diluted 1:5 with negative plasma; LS = pre-mortem (living donor) serum sample; PS = post-mortem serum sample; LS-EP = pre-mortem EDTA plasma sample; PS-EP = post-mortem EDTA plasma sample; *not enough volume of EDTA plasma for analysis; n.a. = not analysed (not enough volume); invalid = not evaluable (cause unknown); SD = standard deviation; n.e. = not evaluable

**Table S7a** – **HCV-RNA** test result summary of spiked paired pre- and post-mortem blood samples.

| Individual Nr. | All samples are diluted 1:5 | **Cobas 6800**  (Roche) | | | **Alinity m**  (Abbott) | | | **Cobas Ampliprep/Cobas TaqMan** (Roche) | | |
| --- | --- | --- | --- | --- | --- | --- | --- | --- | --- | --- |
|  |  | Result  (IU/mL) | Result  (Ct-value) | QC  (Ct-value) | Result  (IU/mL) | Result  (Ct-value) | QC  (Ct-value) | Result  (IU/mL) | Result  (Ct-value) | QC  (Ct-value) |
| 1 | **PS** | 292 | 33.7 | 36.47 | 256 | 25.11 | 20.73 | 207 | 34.5 | 31.9 |
|  | **LS** | 278 | 33.94 | 36.64 | 131 | 26.07 | 21.01 | 385 | 33.7 | 31.9 |
|  | **PS-EP** | 325 | 33.73 | 36.66 | 149 | 25.89 | 21.04 | 234 | 34.2 | 31.8 |
|  | **LS-EP** | 427 | 33.1 | 36.43 | 152 | 25.86 | 20.86 | 448 | 33.7 | 32 |
| 2 | **PS** | 295 | 33.5 | 36.28 | 106 | 26.21 | 21.97 | 258 | 35.2 | 32.1 |
|  | **LS** | 316 | 33.86 | 36.75 | 206 | 25.25 | 20.54 | 256 | 35.3 | 32.1 |
|  | **PS-EP** | 244 | 33.86 | 36.36 | 94 | 26.37 | 22.7 | 190 | 35.4 | 31.9 |
|  | **LS-EP** | 270 | 33.91 | 36.57 | 184 | 25.58 | 20.9 | 305 | 34.3 | 32.1 |
| 3 | **PS** | 183 | 34.49 | 36.57 | 144 | 25.77 | 21.44 | 350 | 34.7 | 32.7 |
|  | **LS** | 343 | 33.25 | 36.26 | 279 | 24.99 | 20.64 | 400 | 33.8 | 32 |
|  | **PS-EP** | 219 | 33.9 | 36.24 | invalid | -- | -- | 306 | 35.3 | 32.4 |
|  | **LS-EP** | 277 | 33.22 | 35.92 | 221 | 25.32 | 20.85 | 549 | 33.9 | 32.5 |
| 4 | **PS** | 224 | 33.34 | 35.72 | 121 | 26.18 | 21.66 | 287 | 34.5 | 32.3 |
|  | **LS** | 308 | 33 | 35.85 | 239 | 25.21 | 20.71 | 347 | 33.8 | 31.8 |
|  | **PS-EP** | 305 | 33.4 | 36.24 | 256 | 25.11 | 20.71 | 305 | 34.1 | 31.9 |
|  | **LS-EP** | 305 | 33.59 | 36.43 | 289 | 24.94 | 20.97 | 367 | 34.7 | 32.8 |
| 5* | **PS** | 218 | 34.78 | 37.12 | 155 | 25.66 | 21.69 | invalid | -- | -- |
|  | **LS** | 327 | 33.51 | 36.45 | 181 | 25.44 | 21 | 323 | 33.7 | 31.6 |
| 6* | **PS** | 260 | 33.43 | 36.03 | 167 | 25.55 | 20.99 | 268 | 34.7 | 32.4 |
|  | **LS** | 333 | 33.74 | 36.7 | 209 | 25.23 | 20.61 | 426 | 33.8 | 32.1 |
| 7 | **PS** | 308 | 33.34 | 36.19 | 295 | 24.74 | 20.42 | 341 | 33.9 | 31.9 |
|  | **LS** | 290 | 33.41 | 36.17 | 173 | 25.5 | 20.9 | 438 | 33.9 | 32.2 |
|  | **PS-EP** | 355 | 33.44 | 36.5 | 225 | 25.13 | 20.84 | 172 | 34.9 | 32.1 |
|  | **LS-EP** | 303 | 33.57 | 36.4 | 220 | 25.16 | 20.85 | 396 | 34 | 32.2 |

**Table S7a** (continued) – **HCV-RNA** test result summary of spiked paired pre- and post-mortem blood samples.

| Individual Nr. | All samples are diluted 1:5 | **Cobas 6800**  (Roche) | | | **Alinity m**  (Abbott) | | | **Cobas Ampliprep/Cobas TaqMan** (Roche) | | |
| --- | --- | --- | --- | --- | --- | --- | --- | --- | --- | --- |
|  |  | Result  (IU/mL) | Result  (Ct-value) | QC  (Ct-value) | Result  (IU/mL) | Result  (Ct-value) | QC  (Ct-value) | Result  (IU/mL) | Result  (Ct-value) | QC  (Ct-value) |
| 8 | **PS** | 225 | 34 | 36.38 | invalid | -- | -- | invalid | -- | -- |
|  | **LS** | 263 | 33.66 | 36.27 | 71 | 26.78 | 21.37 | 424 | 33.7 | 32 |
|  | **PS-EP** | 225 | 34.36 | 36.75 | invalid | -- | -- | invalid | -- | -- |
|  | **LS-EP** | 317 | 33.68 | 36.57 | 157 | 25.64 | 20.69 | 475 | 33.8 | 32.2 |
| 9 | **PS** | 308 | 33.43 | 36.28 | 195 | 25.33 | 21.14 | n.a. | n.a. | n.a. |
|  | **LS** | 369 | 33.6 | 36.72 | 193 | 25.35 | 21.26 | n.a. | n.a. | n.a. |
|  | **PS-EP** | 260 | 34.01 | 36.61 | 178 | 25.46 | 21.08 | n.a. | n.a. | n.a. |
|  | **LS-EP** | 237 | 33.56 | 36.02 | 173 | 25.5 | 21.05 | n.a. | n.a. | n.a. |
| QC | neg. Plasma (dilution plasma) | negative |  | 36.72 | negative | -1.00 | 20.74 | n.a. | n.a. | n.a. |
|  | spiked 1:5 diluted neg. plasma | 322 | 33.92 | 36.83 | 241 | 25.03 | 20.69 | n.a. | n.a. | n.a. |

QC = internal quality control; All samples were diluted 1:5 with negative plasma prior spiking; LS = pre-mortem (living donor) serum sample; PS = post-mortem serum sample; LS-EP = pre-mortem EDTA plasma sample; PS-EP = post-mortem EDTA plasma sample; *not enough volume of EDTA plasma for analysis; n.a. = not analysed (not enough volume); invalid = not evaluable (cause unknown); n.e. = not evaluable

**Table S7b** – Detailed analysis of **HCV-RNA** test results of spiked paired pre- and post-mortem blood samples.

| Individual  Nr. | All samples are diluted 1:5 | **Cobas 6800**  (Roche) | | | **Alinity m**  (Abbott) | | | **Cobas Ampliprep/Cobas TaqMan**  (Roche) | | |
| --- | --- | --- | --- | --- | --- | --- | --- | --- | --- | --- |
|  |  | Result  (IU/mL) | Result  (Ct-value) | QC  (Ct-value) | Result  (IU/mL) | Result  (Ct-value) | QC  (Ct-value) | Result  (IU/mL) | Result  (Ct-value) | QC  (Ct-value) |
| 1 | **PS** | 292 | 33.7 | 36.6 | 256 | 25.1 | 20.7 | 207 | 34.5 | 31.9 |
|  | **LS** | 278 | 33.9 | 36.6 | 131 | 26.1 | 21.0 | 385 | 33.7 | 31.9 |
|  | **Mean** | 285 | 33.8 | 36.6 | 194 | 25.6 | 20.9 | 296 | 34.1 | 31.9 |
|  | **SD** | 9.9 | 0.2 | 0.1 | 88.4 | 0.7 | 0.2 | 125.9 | 0.6 | 0 |
|  |  |  |  |  |  |  |  |  |  |  |
| 1 | **PS-EP** | 325 | 33.7 | 36.7 | 149 | 25.9 | 21.0 | 234 | 34.2 | 31.8 |
|  | **LS-EP** | 427 | 33.1 | 36.4 | 152 | 25.9 | 20.9 | 448 | 33.7 | 32 |
|  | **Mean** | 376 | 33.4 | 36.6 | 151 | 25.9 | 21.1 | 341 | 34.0 | 31.9 |
|  | **SD** | 72.1 | 0.5 | 0.2 | 2.1 | 0.0 | 0.1 | 151.3 | 0.4 | 0.1 |
|  |  |  |  |  |  |  |  |  |  |  |
| 2 | **PS** | 295 | 33.5 | 36.3 | 106 | 26.2 | 22.0 | 258 | 35.2 | 32.1 |
|  | **LS** | 316 | 33.9 | 36.8 | 206 | 25.3 | 20.5 | 256 | 35.3 | 32.1 |
|  | **Mean** | 306 | 33.7 | 36.5 | 156 | 25.7 | 21.3 | 257 | 35.3 | 32.1 |
|  | **SD** | 14.9 | 0.3 | 0.3 | 70.7 | 0.7 | 1.0 | 1.4 | 0.1 | - |
|  | | | | | | | | | | |
| 2 | **PS-EP** | 244 | 33.9 | 36.4 | 94 | 26.4 | 22.7 | 190 | 35.4 | 31.9 |
|  | **LS-EP** | 270 | 33.9 | 36.6 | 184 | 25.6 | 20.9 | 305 | 34.3 | 32.1 |
|  | **Mean** | 257 | 33.9 | 36.5 | 139 | 26.0 | 21.8 | 248 | 34.9 | 32.0 |
|  | **SD** | 18.4 | 0.0 | 0.2 | 63.6 | 0.6 | 1.3 | 81.3 | 0.8 | 0.1 |

| 3 | **PS** | 183 | 34.5 | 36.6 | 144 | 25.8 | 21.4 | 350 | 34.7 | 32.7 |
| --- | --- | --- | --- | --- | --- | --- | --- | --- | --- | --- |
|  | **LS** | 343 | 33.3 | 36.3 | 279 | 25.0 | 20.6 | 400 | 33.8 | 32 |
|  | ***Mean*** | *263* | *33.9* | *36.4* | *212* | *25.4* | *21.0* | *375* | *34.3* | *32.4* |
|  | ***SD*** | *113.1* | *0.9* | *0.2* | *95.5* | *0.6* | *0.6* | *35.4* | *0.6* | *0.5* |
|  |  |  |  |  |  |  |  |  |  |  |
| 3 | **PS-EP** | 219 | 33.9 | 36.2 | invalid | -- | -- | 306 | 35.3 | 32.4 |
|  | **LS-EP** | 277 | 33.22 | 35.9 | 221 | 25.3 | 20.9 | 549 | 33.9 | 32.5 |
|  | ***Mean*** | 248 | 33.56 | 36.1 | 221 | 25.3 | 20.9 | 428 | 34.6 | 32.5 |
|  | ***SD*** | 41.0 | 0.5 | 0.2 | n.e. | n.e. | n.e. | 171.8 | 10.0 | 0.1 |

**Table S7b** (continued) – Detailed analysis of **HCV-RNA** test results of spiked paired pre- and post-mortem blood samples.

| Individual Nr. | All samples are diluted 1:5 | **Cobas 6800**  (Roche) | | | **Alinity m**  (Abbott) | | | **Cobas Ampliprep/Cobas TaqMan**  (Roche) | | |
| --- | --- | --- | --- | --- | --- | --- | --- | --- | --- | --- |
|  |  | Result  (IU/mL) | Result  (Ct-value) | QC  (Ct-value) | Result  (IU/mL) | Result  (Ct-value) | QC  (Ct-value) | Result  (IU/mL) | Result  (Ct-value) | QC  (Ct-value) |
| 4 | **PS** | 224 | 33.34 | 35.7 | 121 | 26.2 | 21.7 | 287 | 34.5 | 32.3 |
|  | **LS** | 308 | 33 | 35.9 | 239 | 25.2 | 20.7 | 347 | 33.8 | 31.8 |
|  | **Mean** | 266 | 33.2 | 35.8 | 180 | 25.7 | 21.2 | 317 | 34.2 | 32.1 |
|  | **SD** | 59.4 | 0.2 | 0.1 | 83.4 | 0.7 | 0.7 | 42.4 | 0.5 | 0.4 |
|  |  |  |  |  |  |  |  |  |  |  |
| 4 | **PS-EP** | 305 | 33.4 | 36.2 | 256 | 25.1 | 20.7 | 305 | 34.1 | 31.9 |
|  | **LS-EP** | 305 | 33.6 | 36.4 | 289 | 24.9 | 21.0 | 367 | 34.7 | 32.8 |
|  | **Mean** | 305 | 33.5 | 36.3 | 273 | 25.0 | 20.8 | 336 | 34.4 | 32.4 |
|  | **SD** | - | 0.1 | 0.1 | 23.3 | 0.1 | 0.2 | 43.8 | 0.4 | 0.6 |

| 5* | **PS** | 218 | 34.8 | 37.1 | 155 | 25.7 | 21.7 | invalid | -- | | -- |
| --- | --- | --- | --- | --- | --- | --- | --- | --- | --- | --- | --- |
|  | **LS** | 327 | 33.5 | 36.5 | 181 | 25.4 | 21 | 323 | 33.7 | | 31.6 |
|  | **Mean** | 273 | 34.2 | 36.8 | 168 | 25.6 | 21.4 | 323 | 34.6 | | 316 |
|  | **SD** | 77.1 | 0.9 | 0.5 | 18.4 | 0.2 | 0.5 | n.e. | n.e. | | n.e. |
|  |  |  |  |  |  |  |  |  | |  |  |
| 6* | **PS** | 260 | 33.4 | 36.03 | 167 | 25.6 | 21.0 | 268 | | 34.7 | 32.4 |
|  | **LS** | 333 | 33.7 | 36.7 | 209 | 25.2 | 20.6 | 426 | | 33.8 | 32.1 |
|  | **Mean** | 297 | 33.6 | 36.4 | 188 | 25.4 | 20.8 | 347 | | 34.3 | 32.3 |
|  | **SD** | 51.6 | 0.2 | 0.5 | 29.7 | 0.2 | 0.3 | 111.7 | | 0.6 | 0.2 |
|  |  |  |  |  |  |  |  |  | |  |  |
| 7 | **PS** | 308 | 33.3 | 36.2 | 295 | 24.7 | 20.4 | 341 | | 33.9 | 31.9 |
|  | **LS** | 290 | 33.4 | 36.2 | 173 | 25.5 | 20.9 | 438 | | 33.9 | 32.2 |
|  | **Mean** | 299 | 33.4 | 36.2 | 234 | 25.1 | 20.7 | 390 | | 33.9 | 32.1 |
|  | **SD** | 12.7 | 0.1 | 0.0 | 86.3 | 0.5 | 0.3 | 68.6 | | - | 0.2 |
|  |  |  |  |  |  |  |  |  | |  |  |
| 7 | **PS-EP** | 355 | 33.4 | 36.5 | 225 | 25.1 | 20.8 | 172 | | 34.9 | 32.1 |
|  | **LS-EP** | 303 | 33.6 | 36.4 | 220 | 25.2 | 20.9 | 396 | | 34 | 32.2 |
|  | **Mean** | 329 | 33.5 | 36.5 | 223 | 25.2 | 20.9 | 284 | | 34.5 | 32.2 |
|  | **SD** | 36.8 | 0.1 | 0.1 | 3.5 | 0.0 | 0.0 | 158.4 | | 0.6 | 0.1 |

**Table S7b** (continued) – Detailed analysis of **HCV-RNA** test results of spiked paired pre- and post-mortem blood samples.

| Individual Nr. | All samples are diluted 1:5 | **Cobas 6800**  **(Fa. Roche)** | | | **Alinity m**  **(Fa. Abbott)** | | | **Cobas Ampliprep/Cobas TaqMan**  **(Fa. Roche)** | | |
| --- | --- | --- | --- | --- | --- | --- | --- | --- | --- | --- |
|  |  | Result  (IU/mL) | Result  (Ct-value) | QC  (Ct-value) | Result  (IU/mL) | Result  (Ct-value) | QC  (Ct-value) | Result  (IU/mL) | Result  (Ct-value) | QC  (Ct-value) |
| 8 | **PS** | 225 | 34 | 36.38 | invalid | -- | -- | invalid | -- | -- |
|  | **LS** | 263 | 33.66 | 36.27 | 71 | 26.78 | 21.37 | 424 | 33.7 | 32 |
|  | **Mean** | 244 | 33.83 | 36.33 | 71 | 26.78 | 21.37 | 424 | 33.70 | 32.00 |
|  | **SD** | 26.87 | 0.24 | 0.08 | n.e. | n.e. | n.e. | n.e. | n.e. | n.e. |
|  |  |  |  |  |  |  |  |  |  |  |
| 8 | **PS-EP** | 225 | 34.36 | 36.75 | invalid | -- | -- | invalid | -- | -- |
|  | **LS-EP** | 317 | 33.68 | 36.57 | 157 | 25.64 | 20.69 | 475 | 33.8 | 32.2 |
|  | **Mean** | 271 | 34.02 | 36.66 | 157 | 25.64 | 20.69 | 475 | 33.80 | 32.20 |
|  | **SD** | 65.05 | 0.48 | 0.13 | n.e. | n.e. | n.e. | n.e. | n.e. | n.e. |

| 9 | **PS** | 308 | 33.43 | 36.28 | 195 | 25.33 | 21.14 | n.a. | n.a. | n.a. |
| --- | --- | --- | --- | --- | --- | --- | --- | --- | --- | --- |
|  | **LS** | 369 | 33.6 | 36.72 | 193 | 25.35 | 21.26 | n.a. | n.a. | n.a. |
|  | **Mean** | 339 | 33.52 | 36.50 | 194 | 25.34 | 21.20 | -- | -- | -- |
|  | **SD** | 43.13 | 0.12 | 0.31 | 1.41 | 0.01 | 0.08 | -- | -- | -- |
|  |  |  |  |  |  |  |  |  |  |  |
| 9 | **PS-EP** | 260 | 34.01 | 36.61 | 178 | 25.46 | 21.08 | n.a. | n.a. | n.a. |
|  | **LS-EP** | 237 | 33.56 | 36.02 | 173 | 25.5 | 21.05 | n.a. | n.a. | n.a. |
|  | **Mean** | 249 | 33.79 | 36.32 | 176 | 25.48 | 21.07 | -- | -- | -- |
|  | **SD** | 16.26 | 0.32 | 0.42 | 3.54 | 0.03 | 0.02 | -- | -- | -- |

All samples were diluted 1:5 with negative plasma; LS = pre-mortem (living donor) serum sample; PS = post-mortem serum sample; LS-EP = pre-mortem EDTA plasma sample; PS-EP = post-mortem EDTA plasma sample; *not enough volume of EDTA plasma for analysis; n.a. = not analysed (not enough volume); invalid = not evaluable (cause unknown); SD = standard deviation; n.e. = not evaluable

**Table S8a** – **HIV-RNA** test result summary of spiked paired pre- and post-mortem blood samples.

| Individual Nr. | All samples are diluted 1:5 | **Cobas 6800**  (Roche) | | | **Alinity m**  (Abbott) | | | **Cobas Ampliprep/Cobas TaqMan** (Roche) | | |
| --- | --- | --- | --- | --- | --- | --- | --- | --- | --- | --- |
|  |  | Result  (copies/mL) | Result  (Ct-value) | QC  (Ct-value) | Result  (copies/mL) | Result  (Ct-value) | QC  (Ct-value) | Result  (copies/mL) | Result  (Ct-value) | QC  (Ct-value) |
| 1 | **PS** | 141 | 35.41 | 37.26 | 80 | 24.74 | 16.87 | 185 | 31.1 | 27.1 |
|  | **LS** | 137 | 35.42 | 37.24 | 120 | 24.19 | 16.72 | 169 | 31.3 | 27.2 |
|  | **PS-EP** | 142 | 35.54 | 37.4 | 179 | 23.63 | 16.75 | 118 | 32 | 27.3 |
|  | **LS-EP** | 188 | 35.33 | 37.57 | 100 | 24.44 | 16.67 | 187 | 31.1 | 27.1 |
| 2 | **PS** | 124 | 35.56 | 37.24 | 167 | 23.73 | 17.75 | n.a. | n.a. | n.a. |
|  | **LS** | 164 | 35.77 | 37.83 | 92 | 24.56 | 17.38 | 179 | 31.3 | 27.2 |
|  | **PS-EP** | 191 | 34.63 | 36.9 | 164 | 23.75 | 17.88 | 192 | 31.7 | 27.7 |
|  | **LS-EP** | 177 | 35.52 | 37.69 | 171 | 23.69 | 16.55 | 176 | 31.2 | 27.1 |
| 3 | **PS** | 166 | 35.32 | 37.4 | invalid | -- | -- | 107 | 32.8 | 28 |
|  | **LS** | 139 | 35.42 | 37.25 | 117 | 24.22 | 16.58 | n.a. | n.a. | n.a. |
|  | **PS-EP** | 184 | 34.66 | 36.87 | 145 | 23.92 | 18.19 | 162 | 31.3 | 27.1 |
|  | **LS-EP** | 112 | 35.36 | 36.91 | 189 | 24.07 | 16.93 | 344 | 31 | 27.8 |
| 4 | **PS** | 147 | 34.94 | 36.85 | 245 | 23.69 | 17.21 | 72 | 31.9 | 26.5 |
|  | **LS** | 115 | 35.77 | 37.34 | 174 | 24.19 | 17.42 | 146 | 31.3 | 26.9 |
|  | **PS-EP** | 162 | 34.89 | 36.93 | 175 | 24.18 | 17.51 | 111 | 31.7 | 27 |
|  | **LS-EP** | 95 | 36.18 | 37.51 | 81 | 25.31 | 16.87 | 193 | 31.1 | 27.1 |
| 5* | **PS** | 187 | 34.64 | 36.88 | 175 | 23.66 | 18.66 | n.a. | n.a. | n.a. |
|  | **LS** | 94 | 36.01 | 37.32 | 184 | 24.11 | 17.42 | 182 | 31.3 | 27.2 |
| 6* | **PS** | 224 | 34.67 | 37.15 | 152 | 24.38 | 16.97 | 256 | 30.8 | 27.3 |
|  | **LS** | 168 | 35.25 | 37.34 | 149 | 23.88 | 16.58 | 124 | 31.7 | 27.1 |
| 7 | **PS** | 111 | 35.94 | 37.47 | 72 | 24.9 | 18.04 | 151 | 31.4 | 27.1 |
|  | **LS** | 111 | 35.87 | 37.4 | 120 | 24.18 | 16.56 | 154 | 31.3 | 27 |
|  | **PS-EP** | 122 | 35.5 | 37.15 | 137 | 24 | 16.43 | 91 | 32.2 | 27.2 |
|  | **LS-EP** | 55 | 36.85 | 37.43 | 111 | 24.29 | 16.61 | 176 | 31.1 | 27 |

**Table S8a** (continued) – **HIV-RNA** test result summary of spiked paired pre- and post-mortem blood samples.

| Individual Nr. | All samples are diluted 1:5 | **Cobas 6800**  (Roche) | | | **Alinity m**  (Abbott) | | | **Cobas Ampliprep/Cobas TaqMan** (Roche) | | |
| --- | --- | --- | --- | --- | --- | --- | --- | --- | --- | --- |
|  |  | Result  (copies/mL) | Result  (Ct-value) | QC  (Ct-value) | Result  (copies/mL) | Result  (Ct-value) | QC  (Ct-value) | Result  (copies/mL) | Result  (Ct-value) | QC  (Ct-value) |
| 8 | **PS** | 133 | 34.4 | 36.93 | invalid | -- | -- | n.a. | n.a. | n.a. |
|  | **LS** | 103 | 35.91 | 37.34 | 133 | 24.04 | 16.45 | n.a. | n.a. | n.a. |
|  | **PS-EP** | 221 | 34.77 | 37.23 | invalid | -- | -- | n.a. | n.a. | n.a. |
|  | **LS-EP** | 99 | 36.32 | 37.69 | 106 | 24.36 | 16.55 | n.a. | n.a. | n.a. |
| 9 | **PS** | 153 | 35.36 | 37.33 | 128 | 24.09 | 16.89 | n.a. | n.a. | n.a. |
|  | **LS** | 152 | 35.01 | 36.97 | 140 | 23.97 | 18.41 | n.a. | n.a. | n.a. |
|  | **PS-EP** | 141 | 35.27 | 37.12 | 112 | 24.28 | 18.27 | n.a. | n.a. | n.a. |
|  | **LS-EP** | 136 | 35.14 | 36.95 | 198 | 23.49 | 17.6 | n.a. | n.a. | n.a. |
| QC | neg. Plasma (dilution plasma) | *negative* |  | *38* | *negative* | *-1* | *16.58* | n.a. | n.a. | n.a. |
|  | spiked 1:5 diluted neg. plasma | *183* | *35.86* | *38.06* | *173* | *23.68* | *16.5* | n.a. | n.a. | n.a. |

QC = internal quality control; All samples were diluted 1:5 with negative plasma prior spiking; LS = pre-mortem (living donor) serum sample; PS = post-mortem serum sample; LS-EP = pre-mortem EDTA plasma sample; PS-EP = post-mortem EDTA plasma sample; *not enough volume of EDTA plasma for analysis; n.a. = not analysed (not enough volume); invalid = not evaluable (cause unknown); n.e. = not evaluable

**Table S8b** – Detailed analysis of **HIV-RNA** test results of spiked paired pre- and post-mortem blood samples.

| Individual  Nr. | All samples are diluted 1:5 | **Cobas 6800**  (Roche) | | | **Alinity m**  (Abbott) | | | **Cobas Ampliprep/Cobas TaqMan**  (Roche) | | |
| --- | --- | --- | --- | --- | --- | --- | --- | --- | --- | --- |
|  |  | Result  (copies/mL) | Result  (Ct-value) | QC  (Ct-value) | Result  (copies/mL) | Result  (Ct-value) | QC  (Ct-value) | Result  (copies/mL) | Result  (Ct-value) | QC  (Ct-value) |
| 1 | **PS** | 141 | 35.41 | 37.26 | 80 | 24.74 | 16.87 | 185 | 31.1 | 27.1 |
|  | **LS** | 137 | 35.42 | 37.24 | 120 | 24.19 | 16.72 | 169 | 31.3 | 27.2 |
|  | **Mean** | 139 | 35.4 | 37.3 | 100 | 24.5 | 16.8 | 177 | 31.2 | 27.2 |
|  | **SD** | 2.8 | 0.0 | 0.0 | 28.3 | 0.4 | 0.1 | 11.3 | 0.1 | 0.1 |
|  |  |  |  |  |  |  |  |  |  |  |
| 1 | **PS-EP** | 142 | 35.5 | 37.4 | 179 | 23.6 | 16.8 | 118 | 32.0 | 27.3 |
|  | **LS-EP** | 188 | 35.3 | 37.6 | 100 | 24.4 | 16.7 | 187 | 31.1 | 27.1 |
|  | **Mean** | 165 | 35.4 | 37.5 | 139.5 | 24.0 | 16.7 | 152.5 | 31.6 | 27.2 |
|  | **SD** | 32.5 | 0.1 | 0.1 | 55.9 | 0.6 | 0.1 | 48.8 | 0.6 | 0.1 |
|  |  |  |  |  |  |  |  |  |  |  |
| 2 | **PS** | 124 | 35.56 | 37.24 | 167 | 23.73 | 17.75 | n.a. | n.a. | n.a. |
|  | **LS** | 164 | 35.77 | 37.83 | 92 | 24.56 | 17.38 | 179 | 31.3 | 27.2 |
|  | **Mean** | 144 | 35.7 | 37.5 | 129.5 | 24.1 | 17.6 | 179 | 31.3 | 27.2 |
|  | **SD** | 28.3 | 0.15 | 0.42 | 53.0 | 0.6 | 0.3 | n.e. | n.e. | n.e. |
|  | | | | | | | | | | |
| 2 | **PS-EP** | 191 | 34.63 | 36.9 | 164 | 23.75 | 17.88 | 192 | 31.7 | 27.7 |
|  | **LS-EP** | 177 | 35.52 | 37.69 | 171 | 23.69 | 16.55 | 176 | 31.2 | 27.1 |
|  | **Mean** | 184 | 35.1 | 37.3 | 167.5 | 23.7 | 17.2 | 184 | 31.5 | 27.4 |
|  | **SD** | 9.9 | 0.6 | 0.6 | 4.95 | 0.0 | 0.9 | 11.31 | 0.4 | 0.4 |

| 3 | **PS** | 166 | 35.32 | 37.4 | invalid | -- | -- | 107 | 32.8 | 28 |
| --- | --- | --- | --- | --- | --- | --- | --- | --- | --- | --- |
|  | **LS** | 139 | 35.42 | 37.25 | 117 | 24.22 | 16.58 | n.a. | n.a. | n.a. |
|  | ***Mean*** | 152.5 | 35.4 | 37.3 | 117 | 24.2 | 16.6 | 107 | 32.8 | 28 |
|  | ***SD*** | 19.1 | 0.1 | 0.1 | n.e. | n.e. | n.e. | n.e. | n.e. | n.e. |
|  |  |  |  |  |  |  |  |  |  |  |
| 3 | **PS-EP** | 184 | 34.7 | 36.9 | 145 | 23.9 | 18.2 | 162 | 31.3 | 27.1 |
|  | **LS-EP** | 112 | 35.4 | 36.9 | 189 | 24.1 | 16.9 | 344 | 31.0 | 27.8 |
|  | ***Mean*** | 148 | 35.0 | 36.9 | 167 | 24.0 | 17.6 | 253 | 31.2 | 27.5 |
|  | ***SD*** | 50.9 | 0.5 | 0.0 | 31.1 | 0.1 | 0.9 | 128.7 | 0.2 | 0.5 |

**Table S8b** (continued) – Detailed analysis of **HIV-RNA** test results of spiked paired pre- and post-mortem blood samples.

| Individual Nr. | All samples are diluted 1:5 | **Cobas 6800**  (Roche) | | | **Alinity m**  (Abbott) | | | **Cobas Ampliprep/Cobas TaqMan**  (Roche) | | |
| --- | --- | --- | --- | --- | --- | --- | --- | --- | --- | --- |
|  |  | Result  (copies/mL) | Result  (Ct-value) | QC  (Ct-value) | Result  (copies/mL) | Result  (Ct-value) | QC  (Ct-value) | Result  (copies/mL) | Result  (Ct-value) | QC  (Ct-value) |
| 4 | **PS** | 147 | 34.9 | 36.9 | 245 | 23.7 | 17.2 | 72 | 31.9 | 26.5 |
|  | **LS** | 115 | 35.8 | 37.3 | 174 | 24.2 | 17.4 | 146 | 31.3 | 26.9 |
|  | **Mean** | 131 | 35.4 | 37.1 | 210 | 23.9 | 17.3 | 109 | 31.6 | 26.7 |
|  | **SD** | 22.6 | 0.6 | 0.3 | 50.2 | 0.4 | 0.1 | 52.1 | 0.4 | 0.3 |
|  |  |  |  |  |  |  |  |  |  |  |
| 4 | **PS-EP** | 162 | 34.9 | 36.9 | 175 | 24.2 | 17.5 | 111 | 31.7 | 27 |
|  | **LS-EP** | 95 | 36.2 | 37.5 | 81 | 25.3 | 16.9 | 193 | 31.1 | 27.1 |
|  | **Mean** | 128.6 | 35.5 | 37.2 | 128 | 24.7 | 17.2 | 152 | 31.4 | 27.1 |
|  | **SD** | 47.2 | 0.9 | 0.4 | 66.5 | 0.8 | 0.5 | 58.0 | 0.4 | 0.1 |

| 5* | **PS** | 187 | 34.6 | 36.9 | 175 | 23.7 | 18.7 | n.a. | n.a. | | n.a. |
| --- | --- | --- | --- | --- | --- | --- | --- | --- | --- | --- | --- |
|  | **LS** | 94 | 36.0 | 37.3 | 184 | 24.1 | 17.4 | 182 | 31.3 | | 27.2 |
|  | **Mean** | 140.7 | 35.3 | 37.1 | 179.5 | 23.9 | 18.0 | 182 | 31.3 | | 27.2 |
|  | **SD** | 65.5 | 1.0 | 0.3 | 6.4 | 0.3 | 0.9 | n.e. | n.e. | | n.e. |
|  |  |  |  |  |  |  |  |  | |  |  |
| 6* | **PS** | 224 | 34.7 | 37.2 | 152 | 24.4 | 17.0 | 256 | | 30.8 | 27.3 |
|  | **LS** | 168 | 35.3 | 37.3 | 149 | 23.9 | 16.6 | 124 | | 31.7 | 27.1 |
|  | **Mean** | 196 | 35.0 | 37.2 | 150.5 | 24.1 | 16.8 | 190 | | 31.3 | 27.2 |
|  | **SD** | 39.6 | 0.4 | 0.1 | 2.1 | 0.4 | 0.3 | 93.3 | | 0.6 | 0.1 |
|  |  |  |  |  |  |  |  |  | |  |  |
| 7 | **PS** | 111 | 35.9 | 37.5 | 72 | 24.9 | 18.0 | 151 | | 31.4 | 27.1 |
|  | **LS** | 111 | 35.9 | 37.4 | 120 | 24.2 | 16.6 | 154 | | 31.3 | 27.0 |
|  | **Mean** | 111 | 35.9 | 37.4 | 96 | 24.5 | 17.3 | 152.5 | | 31.4 | 27.1 |
|  | **SD** | 0.00 | 0.0 | 0.0 | 33.9 | 0.5 | 1.0 | 2.1 | | 0.1 | 0.1 |
|  |  |  |  |  |  |  |  |  | |  |  |
| 7 | **PS-EP** | 122 | 35.5 | 37.2 | 137 | 24.0 | 16.4 | 91 | | 32.2 | 27.2 |
|  | **LS-EP** | 55 | 36.9 | 37.4 | 111 | 24.3 | 16.6 | 176 | | 31.1 | 27.0 |
|  | **Mean** | 88.3 | 36.2 | 37.3 | 124 | 24.1 | 16.5 | 133.5 | | 31.7 | 27.1 |
|  | **SD** | 47.7 | 1.0 | 0.2 | 18.4 | 0.2 | 0.1 | 60.1 | | 0.8 | 0.1 |

**Table S8b** (continued) – Detailed analysis of **HIV-RNA** test results of spiked paired pre- and post-mortem blood samples.

| Individual Nr. | All samples are diluted 1:5 | **Cobas 6800**  **(Fa. Roche)** | | | **Alinity m**  **(Fa. Abbott)** | | | **Cobas Ampliprep/Cobas TaqMan**  **(Fa. Roche)** | | |
| --- | --- | --- | --- | --- | --- | --- | --- | --- | --- | --- |
|  |  | Result  (copies/mL) | Result  (Ct-value) | QC  (Ct-value) | Result  (copies/mL) | Result  (Ct-value) | QC  (Ct-value) | Result  (copies/mL) | Result  (Ct-value) | QC  (Ct-value) |
| 8 | **PS** | 133 | 34.4 | 36.9 | invalid | -- | -- | n.a. | n.a. | n.a. |
|  | **LS** | 103 | 35.9 | 37.3 | 133 | 24.0 | 16.5 | n.a. | n.a. | n.a. |
|  | **Mean** | 118 | 35.2 | 37.1 | 133 | 24.0 | 16.5 | n.a. | n.a. | n.a. |
|  | **SD** | 21.2 | 1.1 | 0.3 | n.e. | n.e. | n.e. | n.a. | n.a. | n.a. |
|  |  |  |  |  |  |  |  |  |  |  |
| 8 | **PS-EP** | 221 | 34.8 | 37.2 | invalid | -- | -- | n.a. | n.a. | n.a. |
|  | **LS-EP** | 99 | 36.3 | 37.7 | 106 | 24.4 | 16.6 | n.a. | n.a. | n.a. |
|  | **Mean** | 159.8 | 35.5 | 37.5 | 106 | 24.4 | 16.6 | n.a. | n.a. | n.a. |
|  | **SD** | 86.6 | 1.1 | 0.3 | n.e. | n.e. | n.e. | n.a. | n.a. | n.a. |

| 9 | **PS** | 153 | 35.4 | 37.3 | 128 | 24.1 | 16.9 | n.a. | n.a. | n.a. |
| --- | --- | --- | --- | --- | --- | --- | --- | --- | --- | --- |
|  | **LS** | 152 | 35.0 | 37.0 | 140 | 24.0 | 18.4 | n.a. | n.a. | n.a. |
|  | **Mean** | 152.5 | 35.2 | 37.2 | 134 | 24.0 | 17.7 | n.a. | n.a. | n.a. |
|  | **SD** | 0.7 | 0.2 | 0.3 | 8.5 | 0.1 | 1.1 | n.a. | n.a. | n.a. |
|  |  |  |  |  |  |  |  |  |  |  |
| 9 | **PS-EP** | 141 | 35.3 | 37.1 | 112 | 24.3 | 18.3 | n.a. | n.a. | n.a. |
|  | **LS-EP** | 136 | 35.1 | 37.0 | 198 | 23.5 | 17.6 | n.a. | n.a. | n.a. |
|  | **Mean** | 138.5 | 35.2 | 37.0 | 155 | 23.9 | 17.9 | n.a. | n.a. | n.a. |
|  | **SD** | 3.5 | 0.1 | 0.1 | 60.8 | 0.6 | 0.5 | n.a. | n.a. | n.a. |

All samples were diluted 1:5 with negative plasma; LS = pre-mortem (living donor) serum sample; PS = post-mortem serum sample; LS-EP = pre-mortem EDTA plasma sample; PS-EP = post-mortem EDTA plasma sample; *not enough volume of EDTA plasma for analysis; n.a. = not analysed (not enough volume); invalid = not evaluable (cause unknown); SD = standard deviation; n.e. = not evaluable

**Table S9** - Detailed evaluation of internal QC values in the testing of 24 post-mortem blood samples for HBV DNA (samples had been tested routinely on the already validated Roche CAP/CTM system prior).

| Individual Nr. | Material | **Cobas 6800**  (Roche) | | | | | **Alinity m**  (Abbott) | | | | | | | **Cobas Ampliprep/Cobas TaqMan**  (Roche) | | | |
| --- | --- | --- | --- | --- | --- | --- | --- | --- | --- | --- | --- | --- | --- | --- | --- | --- | --- |
|  |  | Result  (IU/mL) | | Result  (Ct-value) | QC  (Ct-value) | | Result  (IU/mL) | | Result  (Ct-value) | | QC  (Ct-value) | | | Result  (IU/mL) | | Result  (Ct-value) | QC  (Ct-value) |
| 1 | **PS** | negative | | - | 34.23 | | negative | | - | | 28.13 | | | negative | | - | 28.5 |
| 2 | **PS** | negative | | - | 34.94 | | negative | | - | | 27.46 | | | negative | | - | 28.7 |
| 3 | **PS­-EP** | negative | | - | 34.87 | | negative | | - | | 27.77 | | | negative | | - | 28.8 |
| 4 | **PS** | negative | | - | 34.12 | | negative | | - | | 27.20 | | | negative | | - | 28.8 |
| 5 | **PS** | negative | | - | 34.18 | | negative | | - | | 27.44 | | | negative | | - | 28.5 |
| 6 | **PS** | negative | | - | 33.19 | | negative | | - | | 27.43 | | | negative | | - | 29.8 |
| 7 | **PS-EP** | negative | | - | 33.95 | | negative | | - | | 27.96 | | | negative | | - | 30.2 |
| 8 | **PS-EP** | negative | | - | 34.55 | | negative | | - | | 27.53 | | | negative | | - | 28.5 |
| 9 | **PS** | negative | | - | 33.73 | | negative | | - | | 27.31 | | | negative | | - | 29 |
| 10 | **PS** | negative | | - | 33.56 | | negative | | - | | 27.55 | | | negative | | - | 29.1 |
| 11 | **PS-EP** | negative | | - | 34.16 | | negative | | - | | 27.84 | | | negative | | - | 29.2 |
| 12 | **PS** | negative | | - | 33.24 | | negative | | - | | 27.90 | | | negative | | - | 29.7 |
| 13 | **PS** | negative | | - | 33.59 | | negative | | - | | 27.28 | | | negative | | - | 29.4 |
| 14 | **PS-EP** | negative | | - | 34.08 | | negative | | - | | 27.25 | | | negative | | - | 29.2 |
| 15 | **PS** | negative | | - | 34.19 | | negative | | - | | 27.21 | | | negative | | - | 28.8 |
| 16 | **PS-EP** | negative | | - | 33.3 | | negative | | - | | 27.52 | | | negative | | - | 29.1 |
| 17 | **PS** | negative | | - | 34.09 | | negative | | - | | 27.42 | | | negative | | - | 29 |
| 18 | **PS-EP** | negative | | - | 34.45 | | negative | | - | | 27.75 | | | negative | | - | 29 |
| 19 | **PS-EP** | negative | | - | 33.49 | | negative | | - | | 27.38 | | | negative | | - | 29 |
| 20 | **PS** | negative | | - | 33.48 | | negative | | - | | 27.60 | | | negative | | - | 28.9 |
| 21 | **PS** | negative | | - | 33.66 | | negative | | - | | 27.87 | | | negative | | - | 29.2 |
| 22 | **PS** | negative | | - | 34.54 | | negative | | - | | 27.53 | | | negative | | - | 29.1 |
| 23 | **PS** | negative | | - | 33.56 | | negative | | - | | 27.68 | | | negative | | - | 29 |
| 24 | **PS-EP** | negative | | - | 33.88 | | negative | | - | | 27.26 | | | negative | | - | 29.1 |
|  |  |  |  | | |  | |  | |  | |  |  | |  | |  |
|  |  |  | | *Mean* | ***33.96*** | |  | | *Mean* | | ***27.55*** | | |  | | *Mean* | ***29.07*** |
|  |  |  | | *Min.* | ***33.19*** | |  |  | *Min.* | | ***27.20*** | | |  |  | *Min.* | ***28.5*** |
|  |  |  | | *Max.* | ***34.94*** | |  |  | *Max.* | | ***28.13*** | | |  |  | *Max.* | ***30.2*** |
|  |  |  | | *Delta Ct* | ***1.75*** | |  |  | *Delta Ct* | | ***0.93*** | | |  |  | *Delta Ct* | ***1.7*** |
|  |  |  | | *SD* | ***0.49*** | |  |  | *SD* | | ***0.26*** | | |  |  | *SD* | ***0.40*** |
|  |  |  | | *CV %* | ***0.17*** | |  |  | *CV %* | | ***0.07*** | | |  |  | *CV %* | ***0.12*** |

LS = pre-mortem (living donor) serum sample; PS = post-mortem serum sample; LS-EP = pre-mortem EDTA plasma sample; PS-EP = post-mortem EDTA plasma sample;

SD = standard deviation; CV % = coefficient of variation

**Table S10** - Detailed evaluation of internal QC values in the testing of 24 post-mortem blood samples for HCV RNA (samples had been tested routinely on the already validated Roche CAP/CTM system prior).

| Individual Nr. | Material | **Cobas 6800**  (Roche) | | | | | **Alinity m**  (Abbott) | | | | | | | **Cobas Ampliprep/Cobas TaqMan**  (Roche) | | | |
| --- | --- | --- | --- | --- | --- | --- | --- | --- | --- | --- | --- | --- | --- | --- | --- | --- | --- |
|  |  | Result  (IU/mL) | | Result  (Ct-value) | QC  (Ct-value) | | Result  (IU/mL) | | Result  (Ct-value) | | QC  (Ct-value) | | | Result  (IU/mL) | | Result  (Ct-value) | QC  (Ct-value) |
| 1 | **PS** | negative | | - | 36.55 | | negative | | - | | 20.65 | | | negative | | - | 31.6 |
| 2 | **PS** | negative | | - | 36.22 | | negative | | - | | 20.38 | | | negative | | - | 31.7 |
| 3 | **PS­-EP** | negative | | - | 36.28 | | negative | | - | | 20.89 | | | negative | | - | 31.6 |
| 4 | **PS** | negative | | - | 36.37 | | negative | | - | | 21.9 | | | negative | | - | 32.1 |
| 5 | **PS** | negative | | - | 36.07 | | negative | | - | | 20.66 | | | negative | | - | 31.7 |
| 6 | **PS** | negative | | - | 36.32 | | negative | | - | | 21.74 | | | negative | | - | 32.3 |
| 7 | **PS-EP** | negative | | - | 36.19 | | negative | | - | | 21.93 | | | negative | | - | 32.3 |
| 8 | **PS-EP** | negative | | - | 35.88 | | negative | | - | | 20.76 | | | negative | | - | 31.9 |
| 9 | **PS** | negative | | - | 36.46 | | negative | | - | | 21.02 | | | negative | | - | 32.1 |
| 10 | **PS** | negative | | - | 36.69 | | negative | | - | | 22.02 | | | negative | | - | 32.7 |
| 11 | **PS-EP** | negative | | - | 35.92 | | negative | | - | | 22.71 | | | negative | | - | 32.3 |
| 12 | **PS** | negative | | - | 36.67 | | negative | | - | | 22.22 | | | negative | | - | 32.5 |
| 13 | **PS** | negative | | - | 35.78 | | negative | | - | | 20.7 | | | negative | | - | 31.7 |
| 14 | **PS-EP** | negative | | - | 36.14 | | negative | | - | | 20.79 | | | negative | | - | 31.9 |
| 15 | **PS** | negative | | - | 36.27 | | negative | | - | | 20.85 | | | negative | | - | 31.6 |
| 16 | **PS-EP** | negative | | - | 36.48 | | negative | | - | | 20.47 | | | negative | | - | 31.7 |
| 17 | **PS** | negative | | - | 36.66 | | negative | | - | | 20.89 | | | negative | | - | 31.9 |
| 18 | **PS-EP** | negative | | - | 36.21 | | negative | | - | | 20.91 | | | negative | | - | 31.8 |
| 19 | **PS-EP** | negative | | - | 36.76 | | negative | | - | | 22.38 | | | negative | | - | 33.3 |
| 20 | **PS** | negative | | - | 36.13 | | negative | | - | | 20.39 | | | negative | | - | 32.1 |
| 21 | **PS** | negative | | - | 36.26 | | negative | | - | | 20.7 | | | negative | | - | 31.9 |
| 22 | **PS** | negative | | - | 36.01 | | negative | | - | | 20.63 | | | negative | | - | 31.8 |
| 23 | **PS** | negative | | - | 36.16 | | negative | | - | | 20.44 | | | negative | | - | 31.6 |
| 24 | **PS-EP** | negative | | - | 36.23 | | negative | | - | | 21.27 | | | negative | | - | 32.3 |
|  |  |  |  | | |  | |  | |  | |  |  | |  | |  |
|  |  |  | | *Mean* | ***36.28*** | |  | | *Mean* | | ***21.13*** | | |  | | *Mean* | ***32.00*** |
|  |  |  | | *Min.* | ***35.78*** | |  |  | *Min.* | | ***20.38*** | | |  |  | *Min.* | ***31.6*** |
|  |  |  | | *Max.* | ***36.76*** | |  |  | *Max.* | | ***22.71*** | | |  |  | *Max.* | ***33.3*** |
|  |  |  | | *Delta Ct* | ***0.98*** | |  |  | *Delta Ct* | | ***2.33*** | | |  |  | *Delta Ct* | ***1.7*** |
|  |  |  | | *SD* | ***0.26*** | |  |  | *SD* | | ***0.71*** | | |  |  | *SD* | ***0.42*** |
|  |  |  | | *CV %* | ***0.10*** | |  |  | *CV %* | | ***0.15*** | | |  |  | *CV %* | ***0.13*** |

LS = pre-mortem (living donor) serum sample; PS = post-mortem serum sample; LS-EP = pre-mortem EDTA plasma sample; PS-EP = post-mortem EDTA plasma sample;

SD = standard deviation; CV % = coefficient of variation

**Table S11** - Detailed evaluation of internal QC values in the testing of 24 post-mortem blood samples for HIV RNA (samples had been tested routinely on the already validated Roche CAP/CTM system prior).

| Individual Nr. | Material | **Cobas 6800**  (Roche) | | | | | **Alinity m**  (Abbott) | | | | | | | **Cobas Ampliprep/Cobas TaqMan**  (Roche) | | | |
| --- | --- | --- | --- | --- | --- | --- | --- | --- | --- | --- | --- | --- | --- | --- | --- | --- | --- |
|  |  | Result  (copies/mL) | | Result  (Ct-value) | QC  (Ct-value) | | Result  (copies/mL) | | Result  (Ct-value) | | QC  (Ct-value) | | | Result  (copies/mL) | | Result  (Ct-value) | QC  (Ct-value) |
| 1 | **PS** | negative | | - | 36.55 | | negative | | - | | 16.72 | | | n.a. | | - | -- |
| 2 | **PS** | negative | | - | 36.22 | | negative | | - | | 16.27 | | | n.a. | | - | -- |
| 3 | **PS­-EP** | negative | | - | 36.28 | | negative | | - | | 16.56 | | | n.a. | | - | -- |
| 4 | **PS** | negative | | - | 36.37 | | negative | | - | | 17.55 | | | n.a. | | - | -- |
| 5 | **PS** | negative | | - | 36.07 | | negative | | - | | 16.54 | | | n.a. | | - | -- |
| 6 | **PS** | negative | | - | 36.32 | | negative | | - | | 17.63 | | | n.a. | | - | -- |
| 7 | **PS-EP** | negative | | - | 36.19 | | negative | | - | | 17.64 | | | n.a. | | - | -- |
| 8 | **PS-EP** | negative | | - | 35.88 | | negative | | - | | 16.92 | | | n.a. | | - | -- |
| 9 | **PS** | negative | | - | 36.46 | | negative | | - | | 16.65 | | | n.a. | | - | -- |
| 10 | **PS** | negative | | - | 36.69 | | negative | | - | | 17.95 | | | n.a. | | - | -- |
| 11 | **PS-EP** | negative | | - | 35.92 | | negative | | - | | 18.20 | | | n.a. | | - | -- |
| 12 | **PS** | negative | | - | 36.67 | | invalid | | - | | n.e. | | | n.a. | | - | -- |
| 13 | **PS** | negative | | - | 35.78 | | negative | | - | | 16.57 | | | n.a. | | - | -- |
| 14 | **PS-EP** | negative | | - | 36.14 | | negative | | - | | 16.67 | | | n.a. | | - | -- |
| 15 | **PS** | negative | | - | 36.27 | | negative | | - | | 16.50 | | | n.a. | | - | -- |
| 16 | **PS-EP** | negative | | - | 36.48 | | negative | | - | | 16.48 | | | n.a. | | - | -- |
| 17 | **PS** | negative | | - | 36.66 | | negative | | - | | 16.66 | | | n.a. | | - | -- |
| 18 | **PS-EP** | negative | | - | 36.21 | | negative | | - | | 16.72 | | | n.a. | | - | -- |
| 19 | **PS-EP** | negative | | - | 36.76 | | negative | | - | | 18.27 | | | n.a. | | - | -- |
| 20 | **PS** | negative | | - | 36.13 | | negative | | - | | 16.65 | | | n.a. | | - | -- |
| 21 | **PS** | negative | | - | 36.26 | | negative | | - | | 16.48 | | | n.a. | | - | -- |
| 22 | **PS** | negative | | - | 36.01 | | negative | | - | | 16.56 | | | n.a. | | - | -- |
| 23 | **PS** | negative | | - | 36.16 | | negative | | - | | 16.23 | | | n.a. | | - | -- |
| 24 | **PS-EP** | negative | | - | 36.23 | | negative | | - | | 16.86 | | | n.a. | | - | -- |
|  |  |  |  | | |  | |  | |  | |  |  | |  | |  |
|  |  |  | | *Mean* | ***36.28*** | |  | | *Mean* | | ***16.93*** | | |  | | *Mean* | -- |
|  |  |  | | *Min.* | ***35.78*** | |  |  | *Min.* | | ***16.23*** | | |  |  | *Min.* | -- |
|  |  |  | | *Max.* | ***36.76*** | |  |  | *Max.* | | ***18.27*** | | |  |  | *Max.* | -- |
|  |  |  | | *Delta Ct* | ***0.98*** | |  |  | *Delta Ct* | | ***2.04*** | | |  |  | *Delta CT* | -- |
|  |  |  | | *SD* | ***0.26*** | |  |  | *SD* | | ***0.61*** | | |  |  | *SD* | -- |
|  |  |  | | *CV %* | ***0.095*** | |  |  | *CV %* | | ***0.104*** | | |  |  | *CV %* | -- |

LS = pre-mortem (living donor) serum sample; PS = post-mortem serum sample; LS-EP = pre-mortem EDTA plasma sample; PS-EP = post-mortem EDTA plasma sample;

SD = standard deviation; CV % = coefficient of variation
